# Biotechnological Production, Isolation and Characterisation of (2*R* ,3*S* )‐2,3‐Dihydroxy‐2,3‐Dihydrobenzoate

**DOI:** 10.1111/1751-7915.70228

**Published:** 2025-09-17

**Authors:** Martina Kiel, Israel Barrantes, Dietmar H. Pieper, Karl‐Heinrich Engesser

**Affiliations:** ^1^ Institute of Sanitary Engineering, Water Quality and Solid Waste Management University of Stuttgart Stuttgart Germany; ^2^ Microbial Interactions and Processes Research Group Helmholtz Centre for Infection Research Braunschweig Germany

**Keywords:** biotransformation, chiral building blocks, cis‐2,3‐dihydroxy‐2,3‐dihydrobenzoate, cis‐cyclohexadienediols, p‐cumate 2,3‐dioxygenase

## Abstract

Bacterial Rieske non‐heme iron oxygenases catalyse the transformation of a wide range of aromatic compounds to vicinal *cis*‐dihydrodiols. Such compounds have been successfully applied in chemoenzymatic synthetic routes for, for example, pharmaceuticals, natural products and polymers. In the case of benzoate, only (1*S*,2*R*)‐*cis*‐1,2‐dihydroxy‐2‐hydrobenzoate is readily accessible via enzymatic transformation, but not the regioisomeric *cis*‐2,3‐dihydroxy‐2,3‐dihydrobenzoate (2,3‐DD) or *cis*‐3,4‐dihydroxy‐3,4‐dihydrobenzoate. While trace amounts of putative *cis*‐2,3‐DD have been obtained before by using *p*‐cumate 2,3‐dioxygenase (PCDO) or a combination of chlorobenzene dioxygenase and nitrilase, none of these approaches enabled its production and isolation at a greater scale for potential use as a chiral building block in organic synthesis. We here provide a protocol for biotransformation of benzoate yielding (2*R*,3*S*)‐2,3‐dihydroxy‐2,3‐dihydrobenzoate using the PCDO of *Pseudomonas citronellolis* strain EB200 with negligible formation of side products. An isolation procedure suitable for production of the 2,3‐DD sodium salt monohydrate at high purity (> 95%) at a gram scale, and a comprehensive characterisation of this novel metabolite is given.

## Graphical abstract

Several strategies for biotransformation are compared and a comprehensive analytical characterisation of the novel dihydrodiol is given.

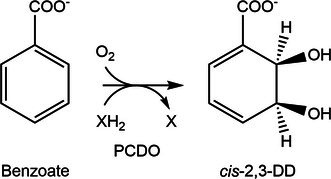

## 1 Introduction

To achieve bacterial aerobic biodegradation of aromatic compounds, typically a limited number of dihydroxybenzenoid compounds such as catechol (1,2‐dihydroxybenzene), protocatechuate 3,4‐dihydroxybenzoate or gentisate (2,5‐dihydroxybenzoate) are produced by so‐called ‘upper pathway enzymes’. These central metabolites are then further metabolised to substrates of the tricarboxylic acid cycle by lower pathway enzymes (Ornston and Stanier 1966; Dagley 1971; Pérez‐Pantoja et al. 2010). The crucial step in the upper pathway—overcoming the stabilising resonance energy of the aromatic ring system—is frequently managed by Rieske non‐heme iron oxygenases (ROs), which typically introduce both atoms of the oxygen molecule into the aromatic ring, forming a *cis*‐dihydrodiol. Since the discovery of this mechanism (Gibson et al. 1968), hundreds of ROs have been identified. ROs are multicomponent enzymes made up of a terminal oxygenase and one or two electron transport proteins. The terminal oxygenase may consist of either a catalytic α‐subunit and a β‐subunit or only an α‐subunit. Electron transport usually from NAD(P)H to the oxygenase is facilitated either by a sole flavoprotein reductase or a combination of a flavoprotein reductase and a ferredoxin. The α subunits are characterised by a conserved Rieske [2Fe‐2S] centre and non‐heme mononuclear iron and are determinants of substrate specificity. Phylogenetic analyses showed that oxygenases can be grouped into families which are characterised by distinct substrate specificities such as phthalate, naphthalene, biphenyl and benzoate dioxygenase types (Nakatsu et al. 1995; Werlen et al. 1996; Duarte et al. 2014). The formation of *cis*‐dihydrodiols from aromatic compounds is unique to prokaryotes. In eukaryotes, oxygenase attack on the aromatic core proceeds via incorporation of only one oxygen atom. The resulting epoxide is either hydrolysed to a *trans*‐dihydrodiol by epoxide hydratase, transformed to a glutathione conjugate nonenzymatically or by glutathione‐*S*‐epoxide transferase or isomerised spontaneously to a ketone that finally undergoes tautomerisation to a phenol (Jerina et al. 1968a, 1968b; Kasperek et al. 1972).

In recent decades, a variety of organic syntheses have made use of *cis*‐dihydrodiols as chiral precursors for pharmaceuticals, polymers, dyes and natural products such as alkaloids, carbasugars and cyclitols (Reed and Hudlicky 2015; Bedard and Hudlicky 2021). Commercially relevant examples include the syntheses of polyphenylene (Ballard et al. 1983) and the HIV‐1 protease inhibitor indinavir (Crixivan, Buckland et al. 1999). Chemical synthesis of arene *cis*‐dihydrodiols, however, is challenging due to the sensitivity of the products to high temperatures and extreme pH values, and due to the need to control chirality during hydroxylation of two adjacent carbon centres. Established methods for the enantioselective dihydroxylation of olefins such as the Sharpless asymmetric dihydroxylation require expensive and toxic catalysts and reagents and are not applicable to aromatic compounds. Few chemical approaches—therefore—have been described for the production of *cis*‐dihydrodiols from arenes, typically as racemates (Jung et al. 1997; Feng et al. 2009; Southgate et al. 2016). In contrast, ROs catalyse the formation of *cis*‐dihydrodiols under mild and environmentally friendly conditions and often in an enantiopure form.

Due to their broad substrate specificity, ROs such as toluene (TDO), naphthalene (NDO), chlorobenzene (CDO), benzoate (BZDO) and biphenyl dioxygenase (BPDO) have been successfully applied to transform a wide range of aromatic and also non‐aromatic compounds to their corresponding *cis*‐dihydrodiols (Johnson 2004). The substrates are dihydroxylated usually in the 2,3‐position relative to the substituent. However, for the simplest aromatic acid, benzoic acid, *cis*‐dihydroxylation in the 2,3‐positions relative to the carboxy group has not been described so far. Unlike the *trans*‐isomer, which is an intermediate of siderophore synthesis in bacteria (Young et al. 1969, 1971), 4,6‐cyclohexadiene‐*cis*‐2,3‐diol‐1‐carboxylate (*cis*‐2,3‐dihydroxy‐2,3‐dihydrobenzoate; 2,3‐DD) does not occur in any known catabolic or anabolic pathways of pro‐ or eukaryotic organisms. Although benzoate degradation via initial 2,3‐dihydroxylation and dehydrogenation to 2,3‐dihydroxybenzoate (2,3‐DHB) seems feasible and the degradation of 2,3‐DHB has been described (Marín et al. 2012), known degradative pathways for benzoate that include an initial attack by a RO proceed exclusively via *ipso*‐(1,2)‐dihydroxylation, forming the (1*S*,2*R*) enantiomer of 3,5‐cyclohexadiene‐*cis*‐1,2‐diol‐1‐carboxylate (*cis*‐1,2‐dihydroxy‐2‐hydrobenzoate; 1,2‐DD; Reiner 1971; Jenkins et al. 1995). Enzymes commonly applied for biotechnological production of *cis*‐dihydrodiols such as TDO and CDO act on hydrophobic substrates, but not on carboxylated aromatics like benzoate (Heald and Jenkins 1996; Yildirim et al. 2006).

As described above, no degradative pathways for benzoate via initial dioxygenase attack in the 2,3‐ or 3,4‐positions are known. In the case of *m*‐ and *p*‐phenoxybenzoate degradation, however, dioxygenation occurs distant to the carboxyl substituent. Here, dioxygenation is directed towards the ether bridge, allowing lateral dioxygenation in the 3,4‐positions and the formation of an instable intermediate that rearranges under cleavage of the ether bond (Engesser et al. 1990). A degradative pathway involving a 2,3‐dioxygenation has been described for *p*‐cumate (DeFrank and Ribbons 1976; Eaton 1996). Transformation of benzoate to 2,3‐DD by *p*‐cumate 2,3‐dioxygenase (PCDO) from *Pseudomonas putida* PL was observed as well (DeFrank and Ribbons 1977). However, transformation was very inefficient, and the low amount of 2,3‐dihydrodiol obtained did not allow for a detailed characterisation of this metabolite. Yildirim and coworkers showed production of 2,3‐DD in a two‐step enzymatic reaction (Yildirim et al. 2006). Starting from benzonitrile, CDO from *Pseudomonas* sp. P51 was applied to produce *cis*‐2,3‐dihydroxy‐2,3‐dihydrobenzonitrile in a whole‐cell biotransformation reaction. The CN moiety was then hydrolysed by nitrilase, yielding 2,3‐DD. Low activity was observed, and besides product verification by HPLC‐MS, no further characterisation was undertaken.

To the best of our knowledge, no further attempts to produce or isolate *cis*‐2,3‐DD have been reported. In this work, we describe the screening for genes encoding enzymes catalysing efficient benzoate‐2,3‐dihydroxylation, their application in homo‐ and heterologous biotransformation of benzoate to 2,3‐DD in a single step, and the isolation and characterisation of the product including the determination of its absolute configuration.

## 2 Materials and Methods

### 2.1 Chemicals

The following chemicals were used in this study: sodium benzoate (Ph. Eur.) and 4‐phenylbenzoic acid, Merck (Darmstadt, Germany); 3‐hydroxybenzoic acid (99%) and 2,3‐dihydroxybenzoic acid, Sigma‐Aldrich (Burlington, USA); sodium salicylate (99%), 4‐ethylbenzoic acid (99%), 4‐isopropylbenzoic acid (99%), 4‐*n*‐propylbenzoic acid (98%) and 4‐*tert*‐butylbenzoic acid (99%), Alfa Aesar (Ward Hill, USA); 4‐methylbenzoic acid, Fluka (Buchs, Switzerland). Other media components and solvents were purchased from Merck, VWR (Darmstadt, Germany), or Carl Roth (Karlsruhe, Germany) and of quality grade ‘pro analysis’ or higher.

### 2.2 Enrichment and Cultivation Conditions

A list of the bacterial strains used in this study is given in Table S1. To enrich and cultivate degraders for substituted benzoates, a variety of compost samples, soils or activated sludge from municipal or industrial wastewater treatment plants were used to inoculate mineral media (MM) containing 1 g L^−1^ KH_2_PO_4_, 3.5 g L^−1^ Na_2_HPO_4_ · 2 H_2_O, 1 g L^−1^ (NH_4_)_2_SO_4_, 0.2 g L^−1^ MgSO_4_ · 7 H_2_O, 0.01 g L^−1^ ferric ammonium citrate (~28% Fe), 0.05 g L^−1^ Ca(NO_3_)_2_ · 4 H_2_O and 1 mL L^−1^ of a trace element solution composed of 0.1 g L^−1^ ZnSO_4_ · 7 H_2_O, 0.03 g L^−1^ MnCl_2_ · 4 H_2_O, 0.3 g L^−1^ H_3_BO_3_, 0.2 g L^−1^ CoCl_2_ · 6 H_2_O, 0.01 g L^−1^ CuCl_2_ · 2 H_2_O, 0.02 g L^−1^ NiCl_2_ · 6 H_2_O and 0.03 g L^−1^ Na_2_MoO_4_ · 2 H_2_O. The respective benzoates were added to final concentrations of 3–5 mM as the sole source of carbon and energy. The cultures were incubated in 500 mL shaking flasks on a rotary shaker at 100 rpm and 30°C and repeatedly transferred to fresh media. Microbial growth was monitored spectrophotometrically (Ultrospec III, Pharmacia Biotech) by determination of the optical density at 546 nm (OD_546_). Pure strains were isolated from enrichment cultures by plating on MM solidified by addition of 15 g L^−1^ agar. To determine substrate spectra, cells pre‐grown with *p*‐ethylbenzoate—or with glucose in the case of ∆*cmtB* mutants—were washed with phosphate buffer (PB) containing 1 g L^−1^ KH_2_PO_4_ and 3.5 g L^−1^ Na_2_HPO_4_ · 2 H_2_O and used to inoculate fresh MM to an OD_546_ of approx. 0.2. Test substrates were added at a concentration of 3 mM, and a control without substrate addition was included. Substrate utilisation was assumed if the OD_546_ increased by at least 100% within 14 days.

### 2.3 DNA Manipulation Techniques

Primers were designed in Primer3 (Untergasser et al. 2012) and ordered from Microsynth (Balgach, Switzerland). A list of the oligonucleotides used in this study is given in Table S2.

PCR was carried out using Taq Polymerase (Bio&Sell, Feucht, Germany) or Phusion polymerase (NEB, Ipswich, USA) when proof‐reading was required. Genomic DNA of bacterial strains was obtained by thermal cracking of cells at 103°C for 10 min and removal of cell fragments by centrifugation at 10,000 ***g*** for 5 min. BOX PCR (Martin et al. 1992) with BOXA1R as the single primer was used for genetic fingerprinting to identify redundant bacterial isolates. Taxonomic assignment of bacterial isolates was carried out by amplification of the 16S rRNA genes with primers 27F and 1492R and identification of closely matching type strain sequences using NCBI BLAST (Altschul et al. 1990). DNA fragments intended for sequencing, restriction digest or ligation were purified using the Monarch PCR and DNA Cleanup Kit (NEB) or the peqGold Gel Extraction Kit (VWR). Restriction enzymes were bought from Fermentas/Thermo Fisher Scientific (Waltham, USA). Ligation reactions were performed using T4 DNA ligase (NEB), and the product was transferred into *E. coli* via electroporation (10 μF, 600 Ω, 1.8 kV). Transformants were selected using 100 μg mL^−1^ ampicillin or 50 μg mL^−1^ kanamycin as adequate. Plasmids were prepared using the NucleoSpin Plasmid Mini kit (Macherey‐Nagel, Düren, Germany). Sanger sequencing was done by Microsynth‐Seqlab (Göttingen, Germany). The plasmids used and constructed in this study are listed in Table S3.

### 2.4 Quantification of Substrates and Biotransformation Products

Culture supernatants were pre‐treated by centrifugation at 21,000 ***g*** for 5 min. HPLC analyses were performed at 210 nm using a SpectraSeries HPLC‐UV/Vis system (Thermo Separation Products) with a ProntoSIL Eurobond C18 column (125 mm * 4.0 mm, 5.0 mm particle size; Bischoff Chromatography, Leonberg, Germany). As mobile phase, a mixture of methanol:water:H_3_PO_4_ = 350:649:1 was used with a flow rate of 1 mL min^−1^. Benzoates and catechols were identified and quantified by comparison with standards prepared from commercially available pure substances. Vicinal *cis*‐dihydrodiols were characterised after heating 900 μL of culture supernatant with 100 μL of 85% ortho‐phosphoric acid for 20 min at 80°C and HPLC analysis of the thereby generated phenolic rearomatisation products. Specifically, 2,3‐DD was quantified by summarising the concentrations of 2‐hydroxybenzoate and 3‐hydroxybenzoate formed during acid‐catalysed rearomatisation, assuming stoichiometric conversion.

### 2.5 Whole Genome Sequencing

A single colony of EB200 was grown overnight in 130 mL MM supplemented with 3 mM 4‐ethylbenzoate as the growth substrate. The cells were harvested by centrifugation and frozen. Bacterial DNA was isolated using the FastDNA Spin Kit for Soil (MP Biomedicals) following the manufacturer's protocol. The quality and concentration of extracted DNA were verified on a Qubit fluorometer. Libraries were prepared according to the instructions from the manufacturer (TruSeq DNA LT Sample Prep Kit, Illumina). Whole genome sequencing was then carried out on a MiSeq Sequencing System (Illumina) and filtered sequences were assembled in SPAdes 3.8.0. The obtained contigs were annotated in PGAP and deposited at GenBank under the accession JBAISM01. Manual analysis to identify ROs involved in aromatic degradative pathways was done in AromaDeg (Duarte et al. 2014). The TYGS server (Meier‐Kolthoff and Göker 2019) was used to query by whole genome comparison if strain EB200 represents a novel or known species. Digital DNA–DNA hybridisation (dDDH) values and confidence intervals were calculated using GGDC 4.0 (recommended settings) (Meier‐Kolthoff et al. 2022).

### 2.6 Generation of Defective Mutants

In‐frame deletions of the genes encoding 2,3‐dihydroxy‐2,3‐dihydro‐*p*‐cumate dehydrogenase (CmtB) and the large subunit of benzoate 1,2‐dioxygenase (BenA) of EB200 were performed as previously described (Schäfer et al. 1994). 600–700 bp fragments flanking the genes to be deleted were amplified, connected by overlap extension PCR and cloned into pK19mobsacB. *E. coli* S17‐1 λpir was transformed with the resulting plasmid, which was then transferred into EB200 by conjugation. Both the donor and the recipient strain were grown in LB medium (containing 50 μg mL^−1^ kanamycin in case of the donor) to the exponential phase, washed twice with 0.9% saline, and adjusted to a concentration of approx. 10^9^ cells/mL. 50 μL aliquots of the suspensions were mixed, applied to a sterile filter paper disk on solid LB medium without antibiotics and incubated overnight. The cell material was then removed from the filter paper by vortexing with 500 μL saline and plated on solid MM with 50 μg mL^−1^ kanamycin and with glucose as the growth substrate. Since pK19mobsacB cannot replicate in pseudomonads, growth of EB200 on media supplemented with kanamycin could occur only after integration of the plasmid into the chromosome. To facilitate subsequent elimination of the plasmid by a second double‐crossover event, the transformed EB200 cells were plated on NSLB agar with 15% saccharose (Hmelo et al. 2015). The colonies obtained were tested for successful deletion of *cmtB* or *benA* by PCR and sequencing of the amplicons. The double mutant EB200 ∆*benA* ∆*cmtB* was generated by subjecting the single mutant EB200 ∆*cmtB* to knock‐out of the BenA gene.

### 2.7 Cloning and Expression of *p*‐Cumate 2,3‐Dioxygenase Genes

A 3.2 kb fragment containing PCDO subunit genes *cmtAa*, *cmtAb* and *cmtAc*, as well as a 0.4 kb fragment containing *cmtAd*, was amplified separately and connected by overlap extension PCR. As expression vector pT7‐5 was chosen, which has been successfully applied for heterologous expression of the four‐component NDO in plasmid pDTG141 (Suen 1991). The NDO genes were removed from pDTG141 by restriction with EcoRI, MluI and HindIII, yielding the linearised backbone of pT7‐5 after gel purification. By ligation with the 3.6 kb overlap construct containing all four components of *cmtA*, expression plasmid pT7‐5(EB200_cmtA) was created. The identity of the insert was verified by sequencing.

Analogously, to construct plasmids pUC118(EB200_cmtA) and pUC118(F1_cmtA), cumene dioxygenase genes were excised from plasmid pIP107D (Aoki et al. 1996) by restriction with EcoRI and HindIII, followed by ligation of *cmtA* overlap constructs from strain EB200 or F1, respectively.

### 2.8 Whole‐Cell Biotransformations

To screen for benzoate 2,3‐dihydroxylating activity, environmental isolates were grown with 5 mM of their respective *p*‐alkylated enrichment substrates, washed with PB and suspended in PB to yield an OD_546_ of 5. Biotransformation was initiated by the addition of 1 mM benzoate.

The production strain EB200 ∆*benA* ∆*cmtB* was grown with 10 mM glucose to an OD_546_ of 1.5–2. Optionally, the culture was supplemented with 3 mM 4‐ethylbenzoate to improve induction of the *p*‐cumate operon. *E. coli* BL21(DE3) was transformed with pT7‐5(EB200_cmtA) and grown overnight in LB medium containing 100 μg mL^−1^ ampicillin. 500 μL of this pre‐culture were then used to inoculate 50 mL of fresh medium. The culture was grown to an OD_600_ of 0.5–0.7, induced with 0.2 mM IPTG and further incubated at room temperature or 30°C for 2–20 h. As a third option, 50 μL of the overnight culture were used to inoculate 50 mL of the autoinduction medium ZYM‐5052 (Studier 2005) supplemented with 100 μg mL^−1^ ampicillin and 10 mg L^−1^ ferric ammonium citrate, which was then incubated for 48 h at room temperature. The induced cells were harvested by centrifugation at 7000 g, washed once with PB and resuspended in 10 mL PB yielding an OD_546_ of approx. 10. Benzoate (5 mM) and glucose (10 mM) for the regeneration of reduction equivalents were added to the resting cell suspensions. Transformation of benzoate was performed at 30°C on a rotary shaker and monitored by HPLC. After fermentation, cells were pelleted by centrifugation and the supernatant was stored at −20°C.

Larger batches intended for isolation and purification of 2,3‐DD were grown to the stationary phase in 500 mL of autoinduction medium ZYM‐5052 in stirred 5 L screw cap bottles at 25°C ± 2.5°C. For these experiments, either BL21(DE3) or Rosetta 2(DE3) (Merck‐Novagen) cells were used, the latter of which were additionally supplemented with 34 μg mL^−1^ chloramphenicol to stabilise the pRARE2 plasmid. After washing with PB, an OD_546_ of approximately 20 was established, and biotransformation at 30°C was initiated and maintained by repeated addition of 5 mM benzoate and 5 mM glucose according to substrate consumption as indicated by HPLC analyses. Oxygen availability was increased by transfer of the suspension to a fresh flask after transformation of 10 mM of benzoate. After transformation for 5–6 h, cells were pelleted by centrifugation and separated from culture supernatants, resuspended in 50 mL PB containing 10 mM of glucose, stored on a rotary shaker at 30°C overnight and reused for biotransformation of benzoate on the following day in fresh PB.

### 2.9 Product Isolation and Purification

Cell‐free culture supernatants were concentrated under reduced pressure by a factor of approx. 10, saturated with NaCl and adjusted to pH 2–2.5 in an ice bath using HCl. After up to four extractions with ethyl acetate (EtOAc) or 1‐butanol (BuOH), the organic and aqueous phases were separated by centrifugation. The organic fractions were combined and re‐extracted once with dilute NaOH. During re‐extraction, the pH of the aqueous phase was monitored and further NaOH was added as necessary for neutralisation. After phase separation supported by centrifugation, the aqueous phase was reduced in a rotational evaporator, followed by further concentration under a nitrogen flow if necessary to give a concentration of approx. 500 mM 2,3‐DD. After mixing with ethanol (EtOH) or isopropyl alcohol (iPrOH), the sodium salt of 2,3‐DD precipitated overnight at room temperature. The supernatant was removed with a pipette and the crystals were dried over silica gel. The potassium salt was prepared analogously by re‐extraction into KOH solution.

### 2.10 Analytical Characterisation of 2,3‐DD

Unless stated otherwise, 2,3‐DD sodium salt monohydrate purified by BuOH extraction and precipitation from water/EtOH mixtures was used for analyses. LC–MS–MS data were obtained from a 10 μg mL^−1^ solution in 50% EtOH using a Quattro Premier mass spectrometer (Micromass Technologies, Manchester, UK) in negative ionisation mode. HR‐MS data were obtained using an Exactive Plus Orbitrap MS (Thermo Fisher Scientific) with electrospray ionisation in negative mode. ^1^H NMR, ^13^C NMR, and HSQC spectra were recorded in D_2_O in an Avance III HD 700 spectrometer (Bruker, Billerica, USA) at 700 MHz. Optical rotation was determined in H_2_O using a P8000‐T80 polarimeter (A.KRÜSS Optronic, Hamburg, Germany) at 20°C and 589 nm. IR absorption was determined via attenuated total reflectance IR spectroscopy of the crystals using an ALPHA FT‐IR spectrometer (Bruker). Crystals suitable for X‐ray crystallography were grown in 200 μL of a 500 mM aqueous solution of the sodium salt by vapour diffusion of EtOH or by layering 400 μL of a 125 mM solution of the potassium salt in 75% iPrOH with 200 μL of pure iPrOH. The vials were left undisturbed to facilitate diffusion and crystal growth over the course of several days.

### 2.11 Spectroscopic Data of 2,3‐DD

^1^H NMR (700 MHz, D_2_O, 23°C): δ 4.324 (1H, dd, *J* = 5.7, 1.1 Hz, H‐2), 4.412 (1H, dt, *J* = 5.5, 2.6 Hz, H‐3), 5.933 (1H, ddt, *J* = 9.6, 2.4, 1.1 Hz, H‐4), 6.039 (1H, ddd, J = 9.6, 5.5, 2.6 Hz, H‐5), 6.728 (1H, d, *J* = 5.4 Hz, H‐6) (Figure S1). The spectrum was consistent with a simulated ^1^H NMR spectrum of 2,3‐DD (Figure S2) and could be clearly distinguished from that of the regioisomeric 1,2‐DD (simulated: Figure S3; empirical data: Reiner and Hegeman 1971; Jenkins et al. 1995; Sun et al. 2008).

^13^C NMR (175 MHz, D_2_O, 23°C): δ 65.6 (C‐2), 70.4 (C‐3), 123.7 (C‐5), 130.9 (C‐6), 133.7 (C‐1), 134.6 (C‐4), 174.6 (COOH) (Figure S4).

HSQC spectrum cf. Figure S5.

Optical rotation (H_2_O, 589 nm, 20°C, β = 5.99 mg mL^−1^): [*α*] = 61.1° mL g^−1^ dm^−1^.

UV/Vis (H_2_O): *λ*_max_(*ε*) = 284 nm (5844 L mol^−1^ cm^−1^) (Figure S6).

LC–MS–MS: m/z: 155 [M^−^], 111 [M^−^—CO_2_], 93 [M^−^—CO_2_–H_2_O], 83.

Exact mass: expected: 155.0350 g mol^−1^; found: 155.0350 g mol^−1^.

IR absorption: *ν*_max_ in cm^−1^: 3294 (OH), 3047 and 2906 (C—H), 1649 (C=C) and 1543 (COO^−^) (Figure S7).

## 3 Results

### 3.1 Enrichment and Screening of Benzoate 2,3‐Dihydroxylating Strains

**FIGURE 1:**
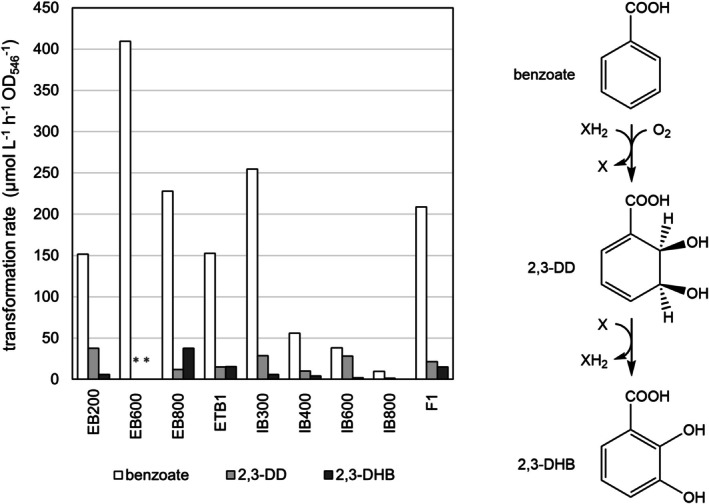
Benzoate dioxygenating activity in bacterial isolates after growth on *p*‐alkylated benzoates. Left side: Conversion rates of benzoate and formation rates of 2,3‐DD and 2,3‐DHB in resting cells of bacterial isolates after growth with 4‐ethylbenzoate or *p*‐cumate (*n* = 1). Samples were taken immediately after addition of benzoate and at *t* = 30 min. *: In EB600, both 2,3‐DD and 2,3‐DHB were detected in trace amounts only in the start sample. Neither benzoate nor metabolites were detectable after 30 min, thus no formation rates for the dihydroxylated metabolites were calculated. Right side: Transformation of benzoate to 2,3‐DHB via *cis*‐2,3‐DD. X, NAD^+^ or NAPH^+^; XH_2_, NADH or NADPH.

Microorganisms were isolated from the environment using substrate analogues of benzoate known to encourage an initial RO attack on the 2,3‐position relative to the carboxy group, that is, *p*‐cumate and other *p*‐alkylated benzoates with *para*‐substituents of varying sizes. Enrichment cultures were successfully set up with 4‐methylbenzoate, 4‐ethylbenzoate and *p*‐cumate (4‐isopropylbenzoate), and a total of 16 pure strains were isolated. These isolates, as well as known 4‐ethylbenzoate and *p*‐cumate degraders available in local strain collections (see Table S1), were screened for transformation of benzoate using resting cells pre‐grown with the respective enrichment substrates. Benzoate transformation rates and production rates for 2,3‐dihydroxylated metabolites are shown in Figure 1 for the 9 strains where a decrease in the benzoate concentration in culture supernatants and excretion of either 2,3‐DHB or suspected 2,3‐DD (inferred by the formation of 2‐ and 3‐hydroxybenzoic acid upon heating the supernatants at acidic pH) was observed. These comprised exclusively 4‐ethylbenzoate and *p*‐cumate degrading strains (Table S1). The 4‐methylbenzoate degraders transformed benzoate, but none produced 2,3‐dihydroxylated metabolites. Instead, traces of 2‐hydroxybenzoate and phenol were found after heating with phosphoric acid, in accordance with earlier observations that 4‐methylbenzoate is typically degraded via 1,2‐dioxygenation by benzoate 1,2‐dioxygenases (Worsey and Williams 1975; Harayama et al. 1991). Benzoate transformation rates of the strains differed widely, and in most cases, less than a third of the transformed benzoate was detectable as 2,3‐dihydroxylated metabolites. The remainder was assumedly transformed beyond the catechol stage either by enzymes of the *p*‐cumate pathway or via other degradative pathways for benzoate.

In addition to *Pseudomonas putida* strain F1, isolates EB800, IB300, IB600 and IB800 were assigned to moderately pathogenic (biosafety level 2) species according to German regulation (TRBA 466) by comparison of their 16S rRNA genes with the NCBI 16S rRNA database (Table S1). Strains EB200, EB600, ETB1 and IB400 were classified as non‐pathogenic (BSL‐1) organisms. Of these, EB200 and EB600 excreted 2,3‐dihydroxylated metabolites even when grown with benzoate as the sole source of carbon (data not shown), owing to either constitutive expression of a *p*‐cumate degradative operon, or induction of *p*‐cumate degradative enzymes by benzoate or its metabolites, or even a hitherto undescribed benzoate degradation pathway via initial 2,3‐dioxygenation. Due to this interesting trait, biosafety considerations, and its undemanding culturability, EB200 was chosen for construction of a biocatalyst for 2,3‐DD production and subject to whole genome sequencing. While strain EB600 exhibited the highest benzoate transforming activity of all strains tested, it was difficult to grow to high cell density, and whole genome sequencing revealed seven incomplete putative *p*‐cumate degradative operons that each contained the α and β subunits of a terminal oxygenase, but not the electron transfer components. As these traits impeded the construction of an efficient biocatalyst from EB600, it was not considered further for the purposes of this study.

### 3.2 Description of *Pseudomonas citronellolis* EB200

The isolate EB200 is a gram‐negative, oxidase‐positive and catalase‐positive bacterium with optimal growth between 25°C and 40°C. It utilises benzoate as well as a wide spectrum of *p*‐substituted benzoates, phenol and benzene as the sole source of carbon and energy, but does not grow on toluene or ethylbenzene. Pairwise comparisons of the genome sequence with those of closely related type strains and calculation of digital DDH values confirmed isolate EB200 to belong to the *Pseudomonas citronellolis* species (dDDH = 73.3, confidence interval = 70.3–76.1 when compared with *Pseudomonas citronellolis* LMG 18378). Further sequence analysis revealed that EB200 possesses both a classical pathway for the degradation of benzoate via initial 1,2‐dihydroxylation (Table S4, MEG7360974 MEG7360979) (Reiner 1971; Jiménez et al. 2002) and a *p*‐cymene type degradative pathway operating via 2,3‐dihydroxylation of intermediary *p*‐cumate (Table S4, MEG7359584‐MEG7359603) (Eaton 1996, 1997). Additionally, genome analysis revealed peripheral pathways for the degradation of anthranilate (Table S4, MEG7360949‐MEG7360951) (Kim et al. 2012), diterpenoids (Table S4, MEG7359769‐MEG7359770, MEG7359779‐MEG7359780 and MEG7359786‐MEG7359787) (Martin and Mohn 2000), phenylacetate (Table S4, MEG7361796‐MEG7361801) (Olivera et al. 1998), 3‐hydroxyphenylacetate (Table S4, MEG7359842‐MEG7359843) (Arias‐Barrau et al. 2005), 3‐hydroxyphenylpropionate (Table S4, MEG7362858) (Díaz et al. 2001), indole 3‐acetic acid (Table S4, MEG7362785‐MEG7362790) (Leveau and Gerards 2008), of 4‐methylphenol via 4‐hydroxybenzoate (Table S4, MEG7363670‐MEG7363670 and MEG7363641) (Hopper 1976; Jiménez et al. 2002), of phenol via a multicomponent phenol hydroxylase system (Table S4, MEG7361570‐MEG7361575) (Shingler et al. 1992), of resorcinol (Table S4, MEG7361292) (Huang et al. 2006) and orcinol (Table S4, MEG7361632) (Ohta and Ribbons 1976). Central metabolic pathways were observed for the degradation of homoprotocatechuate (Table S4, MEG7359876‐MEG7359883) (Arcos et al. 2010), homogentisate (Table S4, MEG7363636‐MEG7363638 and MEG7363640) (Arias‐Barrau et al. 2004), 3‐hydroxyanthranilate (Table S4, MEG7360958‐MEG7360965) (Muraki et al. 2003), 2,3‐dihydroxyphenylpropionate (Table S4, MEG7362859‐MEG7362863) methylhydroquinone (Table S4, MEG7361256) (Tago et al. 2005), hydroxyquinol (Table S4, MEG7361293‐MEG7361294) (Shen et al. 2010), gallate (Table S4, MEG7358775) (Nogales et al. 2005), the intradiol cleavage of protocatechuate (Table S4, MEG7363644‐MEG7363644) (Jiménez et al. 2002) as well as the intradiol (Table S4, MEG7360968‐MEG7360970 and MEG7361568) and extradiol cleavage (Table S4, MEG7361662‐MEG7361668) of catechol (Shingler et al. 1992; Jiménez et al. 2002).

### 3.3 Generation of Knock‐Out Mutants of EB200

Initial benzoate biotransformation studies with 4‐ethylbenzoate‐grown resting cells of EB200 showed that the produced 2,3‐DD was partially dehydrogenated to 2,3‐DHB (Figure 1). A yellow coloration of the suspension suggested subsequent extradiol cleavage. Induction experiments with cells pre‐grown on 10 mM glucose showed immediate growth with *p*‐alkylated benzoates, while growth with benzoate only occurred after a lag phase (Figure S8). Likewise, glucose‐grown cells suspended in mineral growth medium rapidly transformed 4‐ethylbenzoate, whereas benzoate transformation was slow, but increased after one hour, indicating induction of benzoate degrading enzymes (Figure S9). This suggests constitutive expression of the *p*‐cumate degradative pathway in EB200, with benzoate degradation being boosted by an inducible enzyme set. Both further transformation by the *p*‐cumate pathway and induction of a separate benzoate degrading pathway may contribute to a decreased yield and potential contamination of the product by 1,2‐DD, catechols and intermediates of lower degradation pathways, complicating downstream processing and product purification. Engineering of EB200 as a biocatalyst to produce 2,3‐DD therefore required the deletion of both the *p*‐cumate 2,3‐dihydrodiol dehydrogenase (CmtB) and benzoate 1,2‐dioxygenase (BenA) genes (Figure S10). Three defective mutants were created lacking either *benA*, *cmtB* or both, in order to assess their suitability as a production strain for 2,3‐DD, but also to test the eligibility of the *p*‐cumate pathway in EB200 for growth on benzoate as the sole source of carbon and energy.

### 3.4 Pathways for Degradation of Benzoate and *p*‐Cumate in EB200

**FIGURE 2:**
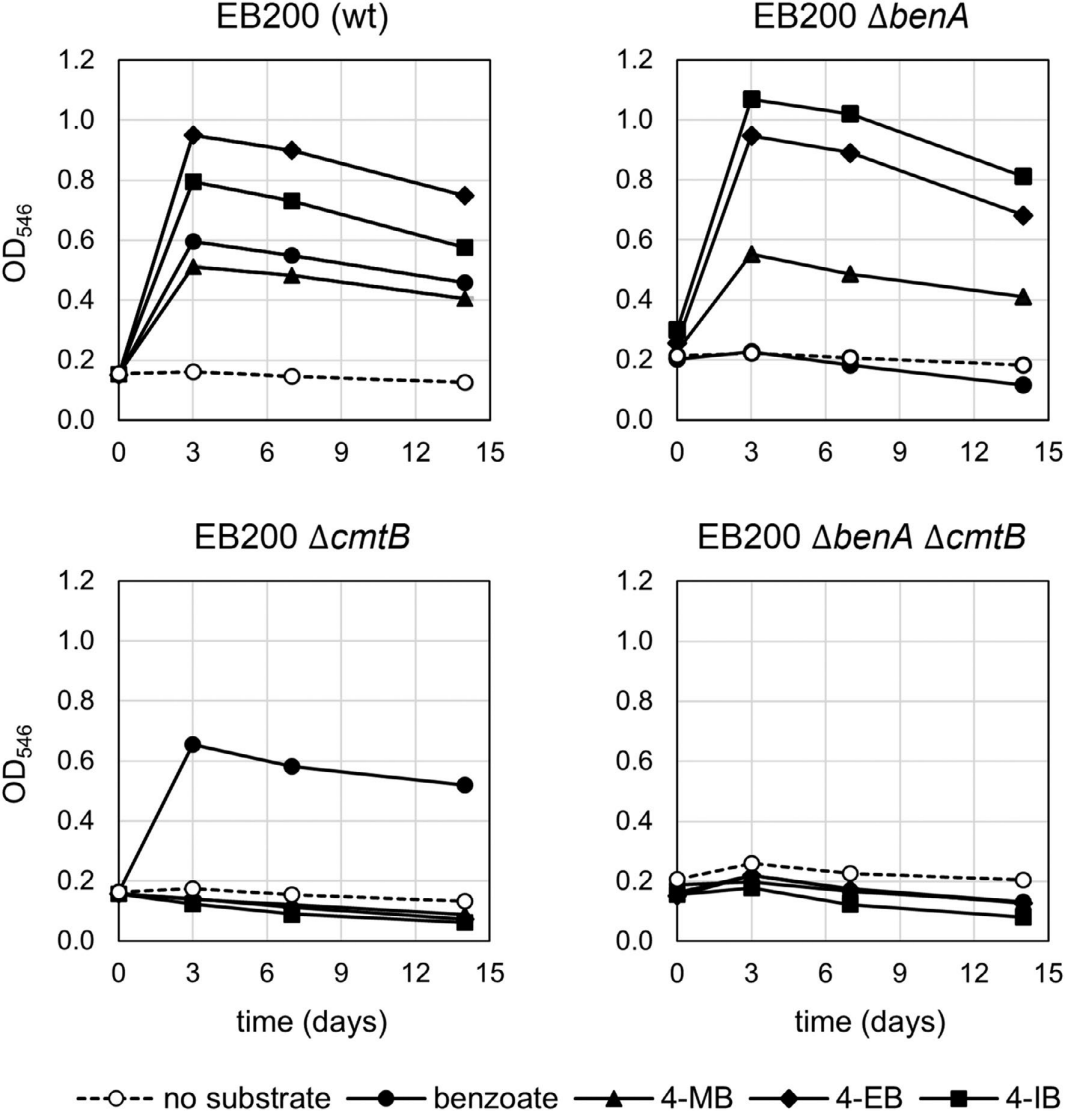
Growth of *P. citronellolis* EB200 wild type and mutants defective in the benzoate and/or *p*‐cumate pathway with benzoate or substituted benzoates as substrates (3 mM substrate) (*n* = 1). Abbreviations: 4‐MB, 4‐methylbenzoate; 4‐EB, 4‐ethylbenzoate; 4‐IB, *p*‐cumate.

Growth experiments with the single and double knock‐out mutants (Figure 2) showed that knock‐out of the *p*‐cumate pathway prevented degradation of *p*‐substituted benzoates, but not of benzoate, while deletion of *benA* prevented only the degradation of unsubstituted benzoate, but not its *p*‐alkylated derivatives. No growth with any of the substrates occurred in the double mutant EB200 ∆*benA* ∆*cmtB*. These results confirm that the pathways for mineralisation of benzoate and *p*‐alkylated benzoates are distinct in EB200.

### 3.5 Production of 2,3‐DD by EB200 ∆ *benA* ∆ *cmtB*

In EB200 wild type glucose‐grown resting cells, only a fraction of transformed benzoate accumulated as 2,3‐dihydroxylated metabolites (Figure S11). Under the applied conditions, roughly 30% of applied benzoate was transformed into 2,3‐DD, with only minor formation of 2,3‐DHB. When cells of the EB200 ∆*cmtB* mutant were used, roughly 50% of consumed benzoate accumulated as 2,3‐DD, and part of the consumed benzoate was obviously routed into the benzoate degradative pathway.

**FIGURE 3:**
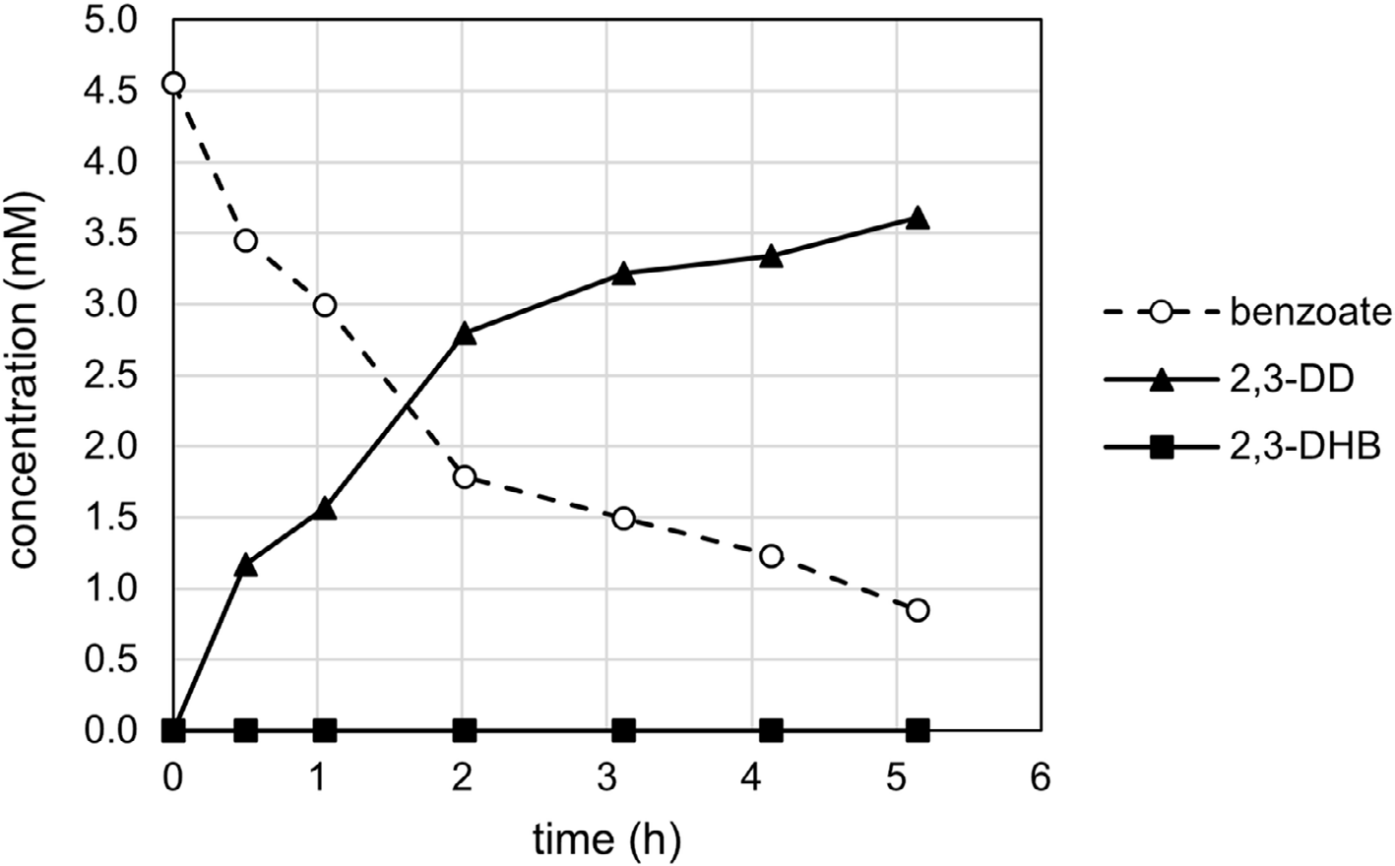
Biotransformation of 5 mM benzoate by glucose‐grown resting cells of the *P. citronellolis* EB200 ∆*benA* ∆*cmtB* mutant at OD_546_ = 10 (*n* = 1).

In contrast, resting cells of EB200 ∆*benA* ∆*cmtB* pre‐grown with glucose transformed benzoate stoichiometrically to 2,3‐DD (Figure 3). No accumulation of unwanted side products such as salicylate, 3‐hydroxybenzoate, 2,3‐DHB, 1,2‐DD or catechol was observed.

**FIGURE 4:**
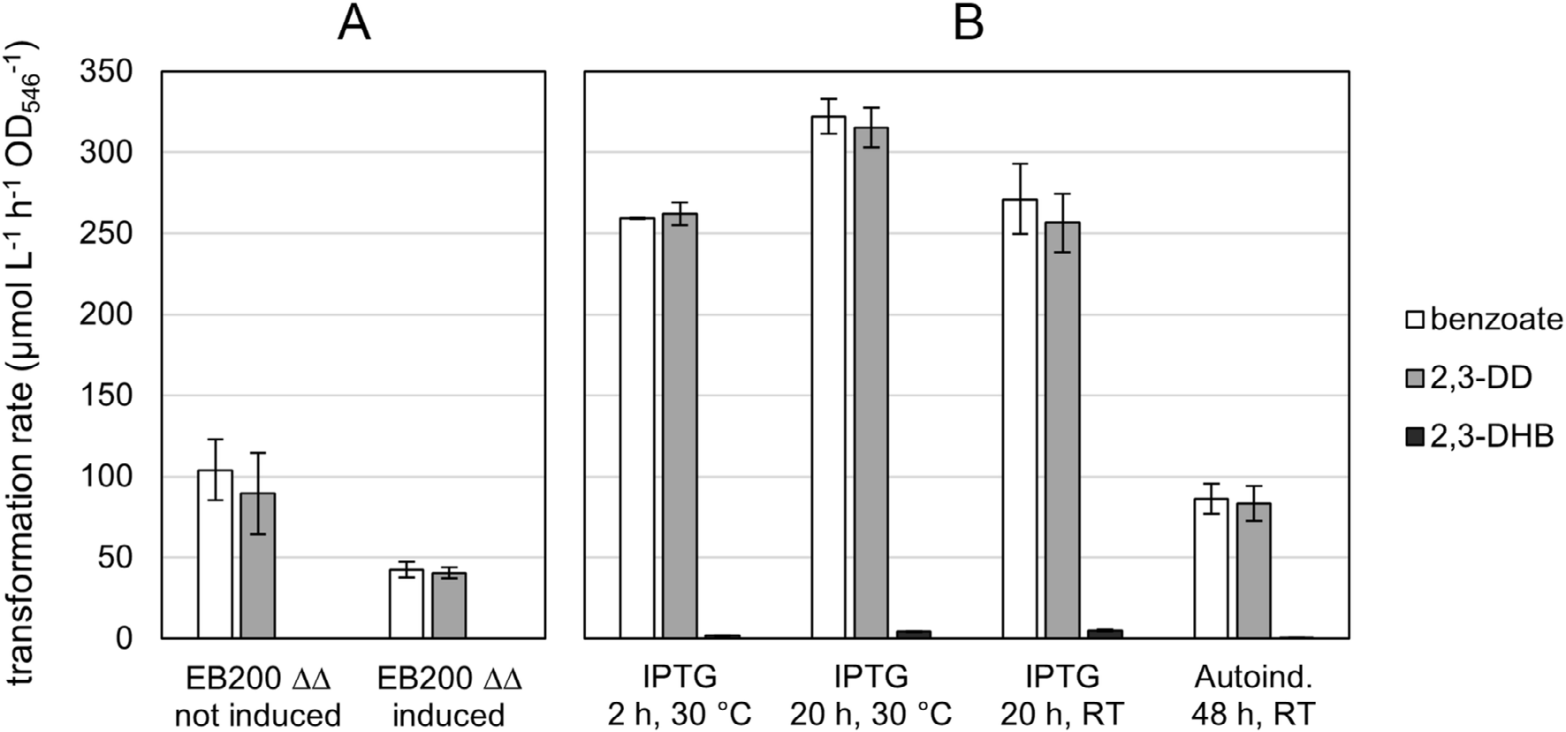
Conversion rates of benzoate and production rates of 2,3‐DD and 2,3‐DHB in resting cell experiments. A: Rates in *P. citronellolis* EB200 ∆*benA* ∆*cmtB* grown with glucose in the absence (EB200 ∆∆ not induced) or presence (EB200 ∆∆ induced) of 3 mM 4‐ethylbenzoate as an inducer. B: Rates in *E. coli* BL21(DE3) pT7‐5(EB200_cmtA) after induction with 0.2 mM IPTG or autoinduction at 30°C or autoinduction at room temperature (RT, 22.5°C ± 2.5°C). Error bars indicate standard deviations (*n* = 3).

However, transformation rates were comparatively low (up to 147 μmol L^−1^ h^−1^ OD_546_^−1^ during the first hour, Table S5), and despite repeated addition of glucose, transformation activity sharply declined when a product concentration of approx. 3 mM was reached. Attempts to improve induction of the *cmt* operon by growing EB200 ∆*benA* ∆*cmtB* in the presence of 3 mM 4‐ethylbenzoate were unsuccessful and yielded even lower transformation rates (Figure 4A).

Unlike the EB200 wild type or ∆*cmtB* mutant, all EB200 ∆*benA* mutants exhibited an altered phenotype with a tendency to form flocs during growth and poor suspendability, which further impeded application in biotransformation experiments.

### 3.6 Production of 2,3‐DD in *E. coli* BL21(DE3)

As a second strategy, plasmids containing PCDO genes were constructed for expression and 2,3‐DD production in an *E. coli* host. Early tests were conducted with expression plasmids derived from pIP107D (Aoki et al. 1996, Table S3), and containing the *cmt*A genes from *P. citronellolis* EB200 or *P. putida* F1, respectively. Expression and biotransformation experiments in *E. coli* JM109 (data not shown) revealed that the transformation rate was three‐fold higher in the case of the EB200‐derived dioxygenase; however, transformation activity declined rapidly after the first hour in both cases. Further optimisation of biotransformation, therefore, focused on CmtA from EB200 and expression in *E. coli* BL21(DE3) using a plasmid under the control of the T7 promoter, pT7‐5(EB200_cmtA).

Conversion of benzoate to 2,3‐DD was analysed after induction with IPTG at room temperature or 30°C for 2 h or 20 h. Highest transformation rates (Figure 4B) were obtained with cells induced for 20 h at 30°C (315 μmol L^−1^ h^−1^ OD_546_^−1^). Statistical significance of the observed differences in 2,3‐DD production rates was estimated by one‐way ANOVA coupled with Tukey post hoc analysis. A significant difference was found between induction conditions 2 h/30°C and 20 h/30°C (*p* = 0.006), and between induction conditions 20 h/RT and 20 h/30°C (*p* = 0.004). Differences between induction conditions 2 h/30°C and 20 h/RT were not significant (*p* = 0.873). The autoinduction approach uses rich growth media supplemented with glucose, glycerol and lactose. Induction of the *lac* operon by lactose occurs only after consumption of glucose, that is late in the growth phase, without further intervention such as adding the inducer at a defined cell density (Studier 2005). Auto‐induced cultures exhibited lower transformation rates in these small‐scale tests, but reached double to three‐fold higher final cell densities, outweighing the difference in OD‐normalised activity while improving convenience of culture handling and monitoring.

**FIGURE 5:**
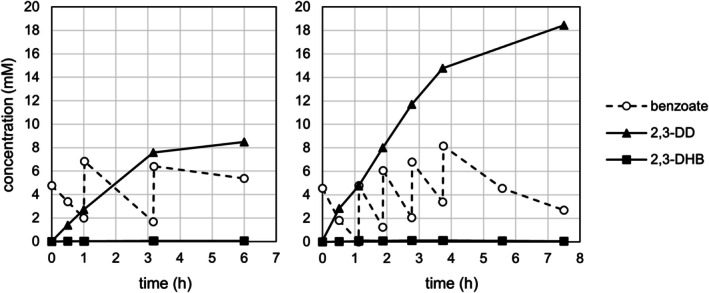
Production of 2,3‐DD in a Rosetta 2(DE3) pT7‐5(EB200_cmtA) fed‐batch culture using cells immediately after autoinduction (left, OD_546_ = 18) and after overnight storage (right, OD_546_ = 17) (representative results). Biotransformation was initiated and maintained by repeated addition of 5 mM benzoate and 5 mM glucose according to substrate consumption as indicated by HPLC analyses.

Larger batches intended for isolation and purification of 2,3‐DD were therefore grown in autoinduction medium ZYM‐5052. For these experiments, either BL21(DE3) or Rosetta 2(DE3) (Merck‐Novagen) cells were used. The latter cell type was applied to boost translation of rare codons in the *E. coli* host, which are contained in the *cmtA* genes at a proportion of up to 6.4%. Overnight storage of the biocatalysts did not negatively affect transformation rates (cf. Figure 5 for representative results) and cells could be recycled for biotransformation on up to four consecutive days. The reason for the improved performance after storage remains unclear and might be attributed to a gradual adaptation of the cells to the fermentation buffer, or to an increased permeability to benzoate. In these cultures, normalised initial benzoate conversion and product formation rates of up to 200 and > 450 μmol L^−1^ h^−1^ OD_546_^−1^ were observed for auto‐induced BL21(DE3) on day 1 and day 2, respectively. Results for Rosetta 2(DE3) were similar at up to 150 and > 450 μmol L^−1^ h^−1^ OD_546_^−1^, ruling out translation of rare codons as a critical factor for biocatalyst activity. Dehydrogenation of the product to 2,3‐DHB was negligible at < 1%. The procedure enabled complete conversion of benzoate when the stored cells were not applied for benzoate transformation in fresh buffers, but to transform residual benzoate in the fermentation supernatant from a previous workday.

### 3.7 Isolation and Purification of 2,3‐DD

The highly hydrophilic nature of 2,3‐DD required protonation to facilitate transfer into organic solvents. Extraction was thus performed at a pH of 2 using an ice bath to delay acid‐catalysed rearomatisation. Considerably higher extraction efficiencies were obtained with BuOH compared to EtOAc as the organic phase. Omission of NaCl led to a high carryover of water into the BuOH phase. A drying step with Na_2_SO_4_ had no effect on the final product quality and was thus not included in the optimised protocol. Further information on the extraction behaviour of 2,3‐DD and undesired buffer components is given as Supporting Information (Appendix I, Table S9).

**FIGURE 6:**
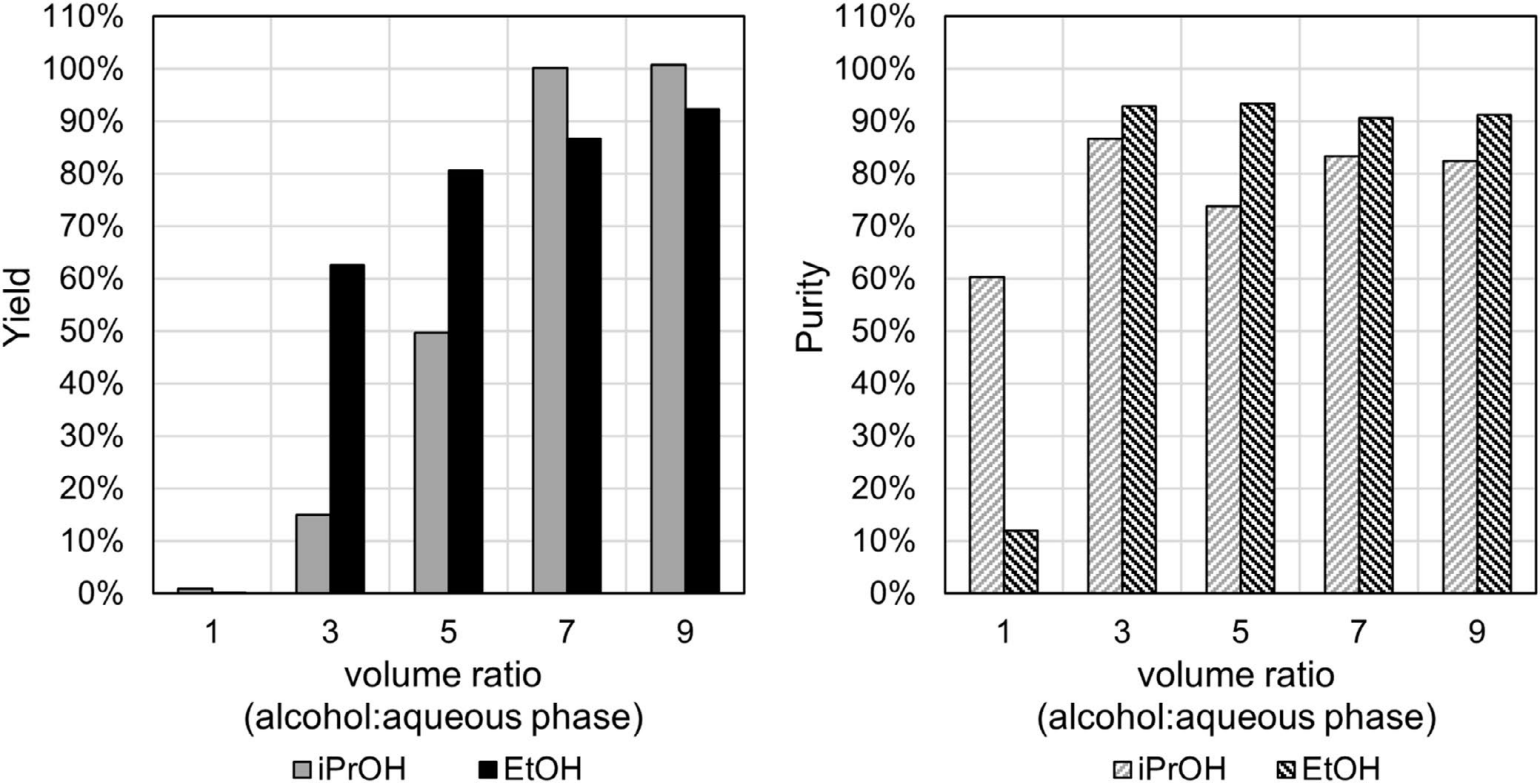
Yield (left) and purity (right) of 2,3‐DD sodium salt monohydrate precipitated from water/alcohol mixtures with increasing proportions of the organic solvent (*n* = 1). Mixtures were prepared by addition of isopropyl alcohol (iPrOH) or ethanol (EtOH) to a 500 mM solution of 2,3‐DD in water at the given volume ratios.

Evaporation even of dried organic phases resulted in rapid aromatisation of the dry residue. A second extraction step with diluted NaOH was thus added to the protocol. At neutral pH, almost complete extraction of 2,3‐DD from the organic into the aqueous phase was observed. The sodium salt of 2,3‐DD was precipitated overnight by addition of EtOH or iPrOH to concentrated aqueous phases, with a volume ratio of EtOH: concentrate = 9:1 giving the optimum results in terms of purity and yield (Figure 6).

No organic contaminants, notably products of enzymatic or chemical rearomatisation, were detectable by HPLC or ^1^H and ^13^C NMR analyses of the final product. The procedure yielded crystals with a purity of 85%–90% (HPLC) referring to water‐free dihydrodiol, corresponding to 94%–99% dihydrodiol monohydrate. The total yield of the optimised procedure was 60%–68%; for yields of single steps cf. Table S6.

A recommended protocol for the production of the stable 2,3‐DD sodium salt monohydrate, summarising the optimal conditions identified in this study, is given as Supporting Information (Appendix II).

### 3.8 Properties of 2,3‐DD

PCDO converts its native substrate to (2*R*,3*S*)‐2,3‐dihydroxy‐2,3‐dihydro‐*p*‐cumate (Taylor et al. 1987). Analogously, unsubstituted benzoate is transformed to (2*R*,3*S*)‐2,3‐dihydroxy‐2,3‐dihydrobenzoate, as verified by X‐ray crystallography of its sodium and potassium salts (Figure S12), respectively. In both cases, crystallisation from water/alcohol mixtures yielded the respective monohydrates of the dihydrodiol. The crystals were stable at room temperature over several months. No evidence for the presence of the (2*S*,3*R*) enantiomer was found.

Solutions of 2,3‐DD in water were stable at 30°C and even at 60°C for several days if a neutral pH was maintained. When heated at acidic pH, the dihydrodiol quickly rearomatised to 3‐hydroxybenzoate (95%) and 2‐hydroxybenzoate (5%) (Figure S13). By titration of an acidified 10 mM solution with NaOH, the pK_a_ value of 2,3‐DD was determined to be 4.3. During this procedure less than 1% of 2,3‐DD was lost due to rearomatisation.

## 4 Discussion

While the methyl ester derivative of 2,3‐DD, methyl (2*R*,3*S*)‐4,6‐cyclohexadiene‐*cis*‐2,3‐diol‐1‐carboxylate, is accessible with a yield of 42% through transformation of benzoic acid methyl ester with TDO (Blacker et al. 1995), this enzyme does not act on benzoate. Microbial access to 2,3‐DD by PCDO from *P. putida* PL (DeFrank and Ribbons 1977) or via hydrolysis of *cis*‐2,3‐dihydroxy‐2,3‐dihydrobenzonitrile (Yildirim et al. 2006) is possible, but inefficient. Here, we demonstrate that the PCDO of *Pseudomonas citronellolis* EB200 can be applied for efficient, single‐step biosynthesis of 2,3‐DD using an *E. coli‐*based expression system.

Various bacterial strains isolated for their capability to mineralise 4‐ethylbenzoate and *p*‐cumate were able to transform benzoate to 2,3‐dihydroxylated metabolites, in contrast to earlier research suggesting that unsubstituted benzoate is a poor substrate for PCDOs (DeFrank and Ribbons 1977). Of these, *P. citronellolis* EB200 transformed benzoate at half the rate observed with *p*‐cumate, and one quarter of the rate observed with 4‐ethylbenzoate (Table S7), and produced 2,3‐DD and 2,3‐DHB even when grown with glucose in the absence of *p*‐alkylated benzoates. It was thus chosen as a donor for PCDO genes as well as for construction of a production strain by knock‐out of its 2,3‐dihydroxy‐2,3‐dihydro‐*p*‐cumate dehydrogenase and benzoate 1,2‐dioxygenase.

Deletion of BZDO also disabled growth of EB200 with benzoate, even though benzoate is transformed by constitutively expressed enzymes of the *p*‐cumate pathway in strain EB200 at least up to the carboxycatechol stage. Yellow coloration of culture supernatants suggested subsequent extradiol (*meta*‐)cleavage of 2,3‐DHB. Unlike other β‐keto acids, the resulting 2‐hydroxy‐3‐carboxymuconic semialdehyde is stable at physiological conditions and requires enzymatic decarboxylation to yield 2‐hydroxymuconic semialdehyde (2‐HMS) (Marín et al. 2012). A respective decarboxylase (*cmtD*) is encoded downstream of *cmtAd*. 2‐HMS is also the first intermediate of the *meta‐*cleavage pathway for catechol, which is present in EB200 and could facilitate further transformation to products that can enter the tricarboxylic acid cycle. The mineralisation of 2,3‐DHB along such a pathway (Figure S14) has been demonstrated before (Ribbons 1966; Ribbons and Senior 1970; Andreoni et al. 1981; Marín et al. 2012) but apparently cannot be achieved in EB200. The reason for this failure is unclear. However, it may be speculated to result from the absence of a 2‐hydroxymuconic semialdehyde dehydrogenase necessary for the mineralisation of 2,3‐DHB from the *p*‐cumate degradative operon in EB200 (see Table S4, MEG7359584‐MEG7359596), whereas a 2‐hydroxy‐6‐oxo‐7‐methylocta‐2,4‐dienoate hydrolase involved in *p*‐cumate degradation is present (see Table S4, MEG7359588). These findings also show that EB200 does not employ a novel pathway for mineralisation of benzoate via 2,3‐DD and 2,3‐DHB, and generally underscore that inference of degradative pathways merely from metabolites detectable in culture media may lead to invalid conclusions.

Although anthranilate 1,2‐dioxygenase is known to also dihydroxylate benzoate in the 1,2‐positions (Eby et al. 2001), no evidence for an activity of the anthranilate degradative pathway (see Table S4, MEG7360949‐MEG7360951) of strain EB200 was found during biotransformation or growth experiments with benzoate in *benA* defective mutants.

Formation of 2,3‐DD from benzoate was achieved both by use of the double knock‐out mutant EB200 ∆*benA* ∆*cmtB* and *E. coli* BL21(DE3) derivatives containing the expression plasmid pT7‐5(EB200_cmtA). However, biotransformation in the EB200‐derived production strain was characterised by low conversion rates that rapidly levelled off. *E. coli* ‐based systems reached both higher transformation rates (exceeding 450 μmol L^−1^ h^−1^ OD_546_^−1^) and higher product concentrations (up to 20 mM) over the course of a working day. Optimisation of biotransformation using a continuously fed and aerated reactor might increase transformation rates and final 2,3‐DD concentrations further but was not tested in this study.

The transformation activity of auto‐induced *E. coli* cells was significantly lower compared to IPTG‐induced cells during preliminary small‐scale tests. Upscaling of 2,3‐DD production did not solely focus on increased transformation rates, but in view of future application in large‐scale processes also considered convenience of cultivation, as well as economic and environmental aspects. Auto‐induced cultures reached higher cell densities compared to their IPTG‐induced counterparts, compensating for differences in specific activity. The simplicity of the autoinduction approach (Studier 2005), which does not require careful monitoring of cell density and addition of IPTG, and the use of lactose as a cheap and environmentally friendly inducer makes it an alternative worth considering for production of 2,3‐DD at larger scales. Interestingly, auto‐induced cells did not lose their activity upon overnight storage in phosphate buffer. Recycling of the biocatalysts further lowered costs and the environmental impact of the biotransformation.

In the absence of 2,3‐dihydroxy‐2,3‐dihydro‐*p*‐cumate dehydrogenase, 2,3‐DD was stable in fermentation supernatants. Unlike recently observed with *cis*‐1,2‐dihydroxy‐1,2‐dihydrobenzene (Wissner et al. 2021), unspecific host enzymes such as glycerol dehydrogenase did not catalyse dehydrogenation of 2,3‐DD to a catechol.

Low extraction coefficients were determined during attempts to isolate *cis*‐2,3‐DD from culture supernatants with EtOAc. As previously observed with *trans*‐2,3‐DD (Franke et al. 2003) and cyclohexane‐1,2‐diol (König et al. 2018), 1‐butanol was a promising alternative for the extraction of *cis*‐2,3‐DD not only in terms of efficiency but also under the aspect of green chemistry (Anastas and Warner 2000), since it can be sustainably produced from biomass at the industrial scale and sufficient quality for extraction purposes (Reetz and König 2021).

Like other cyclohexadiene‐c*is*‐diols, 2,3‐DD is a highly functionalised molecule that offers various options for further transformation (cf. Figure 2 in Hudlicky and Reed 2009; Figure 1 in Griffen et al. 2014) and is therefore of potential interest as a chiral building block in organic syntheses. Specifically, 2,3‐DD contains an exocyclic carbon atom, a useful trait for application in the synthesis of pseudo‐sugars (Fabris et al. 2009). Besides biotechnological application, 2,3‐DD may prove useful in enzymatic studies for the elucidation of bacterial pathways, for example, the benzoate degradative pathway in isolate *Neobacillus* sp. EB600 that also excreted 2,3‐DD and 2,3‐DHB during growth with benzoate. Whether these metabolites are generated by side activities of constitutively expressed *p*‐cumate degradative enzymes as well, or if they are genuine intermediates of a novel pathway for the degradation of benzoate via 2,3‐dihydroxylation, remains yet to be examined.

## Author Contributions

**Martina Kiel:** conceptualization, investigation, funding acquisition, writing – original draft, writing – review and editing. **Israel Barrantes:** investigation, writing – review and editing. **Dietmar H. Pieper:** investigation, writing – review and editing. **Karl‐Heinrich Engesser:** supervision, funding acquisition, resources, writing – review and editing.

## Conflicts of Interest

The authors declare no conflicts of interest.

## Supporting information


**Data S1:** mbt270228‐sup‐0001‐Supinfo.docx.

## Data Availability

Additional data that support the findings of this study are available in the Supporting Information of this article. Genomic data of strain EB200 are publicly available at GenBank under the accession JBAISM01, BioProject PRJNA1076549. Crystallographic data of 2,3‐DD are openly available from the Cambridge Crystallographic Data Centre at www.ccdc.cam.ac.uk/structures, reference numbers CCDC 2380197–2380198. Other data are available from the corresponding author upon reasonable request.

## References

[mbt270228-bib-0001] Altschul, S. F. , W. Gish , W. Miller , E. W. Myers , and D. J. Lipman . 1990. “Basic Local Alignment Search Tool.” Journal of Molecular Biology 215, no. 3: 403–410. 2231712 10.1016/S0022-2836(05)80360-2

[mbt270228-bib-0002] Anastas, P. T. , and J. C. Warner . 2000. “Green Chemistry: Oxford University PressOxford.”

[mbt270228-bib-0003] Andreoni, V. , L. Canonica , E. Galli , C. Gennari , and V. Treccani . 1981. “2,3‐Dihydroxybenzoate Pathway in *Pseudomonas putida* . ^1^H n.m.r. Study on the Ring‐Cleavage Site.” Biochemical Journal 194, no. 2: 607–610. 7306005 10.1042/bj1940607 PMC1162785

[mbt270228-bib-0004] Aoki, H. , T. Kimura , H. Habe , H. Yamane , T. Kodama , and T. Omori . 1996. “Cloning, Nucleotide Sequence, and Characterization of the Genes Encoding Enzymes Involved in the Degradation of Cumene to 2‐Hydroxy‐6‐Oxo‐7‐Methylocta‐2,4‐Dienoic Acid in *Pseudomonas fluorescens* IP01.” Journal of Fermentation and Bioengineering 81, no. 3: 187–196.

[mbt270228-bib-0005] Arcos, M. , E. R. Olivera , S. Arias , G. Naharro , and J. M. Luengo . 2010. “The 3,4‐Dihydroxyphenylacetic Acid Catabolon, a Catabolic Unit for Degradation of Biogenic Amines Tyramine and Dopamine in *Pseudomonas putida* U.” Environmental Microbiology 12, no. 6: 1684–1704. 20482587 10.1111/j.1462-2920.2010.02233.x

[mbt270228-bib-0006] Arias‐Barrau, E. , E. R. Olivera , J. M. Luengo , et al. 2004. “The Homogentisate Pathway: A Central Catabolic Pathway Involved in the Degradation of L‐Phenylalanine, L‐Tyrosine, and 3‐Hydroxyphenylacetate in *Pseudomonas putida* .” Journal of Bacteriology 186, no. 15: 5062–5077. 15262943 10.1128/JB.186.15.5062-5077.2004 PMC451635

[mbt270228-bib-0007] Arias‐Barrau, E. , A. Sandoval , G. Naharro , E. R. Olivera , and J. M. Luengo . 2005. “A Two‐Component Hydroxylase Involved in the Assimilation of 3‐Hydroxyphenyl Acetate in *Pseudomonas putida* .” Journal of Biological Chemistry 280, no. 28: 26435–26447. 15866873 10.1074/jbc.M501988200

[mbt270228-bib-0008] Ballard, D. G. H. , A. Courtis , I. M. Shirley , and S. C. Taylor . 1983. “A Biotech Route to Polyphenylene.” Journal of the Chemical Society, Chemical Communications 17: 954.

[mbt270228-bib-0009] Bedard, K. , and T. Hudlicky . 2021. “Enzymatic Dihydroxylation of Aromatic Compounds: Nature's Unique Reaction and Its Impact on the Synthesis of Natural Products.” In Strategies and Tactics in Organic Synthesis, edited by M. Harmata , 53–97. Elsevier.

[mbt270228-bib-0010] Blacker, A. J. , R. J. Booth , G. M. Davies , and J. K. Sutherland . 1995. “Syntheses of 6β‐Hydroxyshikimic Acid and Its Derivatives.” Journal of the Chemical Society, Perkin Transactions 1 113, no. 22: 2861–2870.

[mbt270228-bib-0011] Buckland, B. C. , S. W. Drew , N. C. Connors , et al. 1999. “Microbial Conversion of Indene to Indandiol: A Key Intermediate in the Synthesis of CRIXIVAN.” Metabolic Engineering 1, no. 1: 63–74. 10935755 10.1006/mben.1998.0107

[mbt270228-bib-0012] Dagley, S. 1971. “Catabolism of Aromatic Compounds by Micro‐Organisms.” Advances in Microbial Physiology 6: 1–46. 4950664 10.1016/s0065-2911(08)60066-1

[mbt270228-bib-0013] DeFrank, J. J. , and D. W. Ribbons . 1976. “The *p*‐Cymene Pathway in *Pseudomonas putida* PL: Isolation of a Dihydrodiol Accumulated by a Mutant.” Biochemical and Biophysical Research Communications 70, no. 4: 1129–1135. 942434 10.1016/0006-291x(76)91020-2

[mbt270228-bib-0014] DeFrank, J. J. , and D. W. Ribbons . 1977. “ *P*‐Cymene Pathway in *Pseudomonas putida* : Initial Reactions.” Journal of Bacteriology 129, no. 3: 1356–1364. 845117 10.1128/jb.129.3.1356-1364.1977 PMC235110

[mbt270228-bib-0015] Díaz, E. , A. Ferrández , M. A. Prieto , and J. L. García . 2001. “Biodegradation of Aromatic Compounds by *Escherichia coli* .” Microbiology and Molecular Biology Reviews 65, no. 4: 523–569. 11729263 10.1128/MMBR.65.4.523-569.2001 PMC99040

[mbt270228-bib-0016] Duarte, M. , R. Jauregui , R. Vilchez‐Vargas , H. Junca , and D. H. Pieper . 2014. “AromaDeg, a Novel Database for Phylogenomics of Aerobic Bacterial Degradation of Aromatics.” Database 2014: 1–12. 10.1093/database/bau118 PMC4250580 25468931

[mbt270228-bib-0017] Eaton, R. W. 1996. “ *P*‐Cumate Catabolic Pathway in *Pseudomonas putida* F1: Cloning and Characterization of DNA Carrying the *Cmt* Operon.” Journal of Bacteriology 178, no. 5: 1351–1362. 8631713 10.1128/jb.178.5.1351-1362.1996 PMC177810

[mbt270228-bib-0018] Eaton, R. W. 1997. “P‐Cymene Catabolic Pathway in *Pseudomonas putida* F1: Cloning and Characterization of DNA Encoding Conversion of *p*‐Cymene to *p*‐Cumate.” Journal of Bacteriology 179, no. 10: 3171–3180. 9150211 10.1128/jb.179.10.3171-3180.1997 PMC179094

[mbt270228-bib-0019] Eby, D. M. , Z. M. Beharry , E. D. Coulter , D. M. Kurtz , and E. L. Neidle . 2001. “Characterization and Evolution of Anthranilate 1,2‐Dioxygenase From *Acinetobacter* sp. Strain ADP1.” Journal of Bacteriology 183, no. 1: 109–118. 11114907 10.1128/JB.183-1.109-118.2001 PMC94856

[mbt270228-bib-0020] Engesser, K. H. , W. Fietz , P. Fischer , P. Schulte , and H.‐J. Knackmuss . 1990. “Dioxygenolytic Cleavage of Aryl Ether Bonds: 1,2‐Dihydro‐1,2‐Dihydroxy‐4‐Carboxybenzophenone as Evidence for Initial 1,2‐Dioxygenation in 3‐ and 4‐Carboxy Biphenyl Ether Degradation.” FEMS Microbiology Letters 69, no. 3: 317–321. 10.1016/0378-1097(90)90087-7 2210344

[mbt270228-bib-0021] Fabris, F. , J. Collins , B. Sullivan , H. Leisch , and T. Hudlicky . 2009. “Investigation of Steric and Functionality Limits in the Enzymatic Dihydroxylation of Benzoate Esters. Versatile Intermediates for the Synthesis of Pseudo‐Sugars, Amino Cyclitols, and Bicyclic Ring Systems.” Organic & Biomolecular Chemistry 7, no. 12: 2619–2627. 19503938 10.1039/b902577b

[mbt270228-bib-0022] Feng, Y. , C.‐Y. Ke , G. Xue , and L. Que . 2009. “Bio‐Inspired Arene *Cis*‐Dihydroxylation by a Non‐Haem Iron Catalyst Modeling the Action of Naphthalene Dioxygenase.” Chemical Communications 1: 50–52. 10.1039/b817222f 19081995

[mbt270228-bib-0023] Franke, D. , V. Lorbach , S. Esser , et al. 2003. “(S,S)‐2,3‐Dihydroxy‐2,3‐Dihydrobenzoic Acid: Microbial Access With Engineered Cells of *Escherichia coli* and Application as Starting Material in Natural‐Product Synthesis.” Chemistry 9, no. 17: 4188–4196. 12953204 10.1002/chem.200204265

[mbt270228-bib-0024] Gibson, D. T. , J. R. Koch , and R. E. Kallio . 1968. “Oxidative Degradation of Aromatic Hydrocarbons by Microorganisms. I. Enzymatic Formation of Catechol From Benzene.” Biochemistry 7, no. 7: 2653–2662. 4298226 10.1021/bi00847a031

[mbt270228-bib-0025] Griffen, J. A. , S. J. Kenwright , S. Abou‐Shehada , S. Wharry , T. S. Moody , and S. E. Lewis . 2014. “Benzoate Dioxygenase From *Ralstonia eutropha* B9—Unusual Regiochemistry of Dihydroxylation Permits Rapid Access to Novel Chirons.” Organic Chemistry Frontiers 1, no. 1: 79–90.

[mbt270228-bib-0026] Harayama, S. , M. Rekik , A. Bairoch , E. L. Neidle , and L. N. Ornston . 1991. “Potential DNA Slippage Structures Acquired During Evolutionary Divergence of *Acinetobacter calcoaceticus* Chromosomal *benABC* and *Pseudomonas putida* TOL pWW0 Plasmid *xylXYZ*, Genes Encoding Benzoate Dioxygenases.” Journal of Bacteriology 173, no. 23: 7540–7548. 1938949 10.1128/jb.173.23.7540-7548.1991 PMC212521

[mbt270228-bib-0027] Heald, S. C. , and R. O. Jenkins . 1996. “Expression and Substrate Specificity of the Toluene Dioxygenase of *Pseudomonas putida* NCIMB 11767.” Applied Microbiology and Biotechnology 45, no. 1–2: 56–62. 8920179 10.1007/s002530050649

[mbt270228-bib-0028] Hmelo, L. R. , B. R. Borlee , H. Almblad , et al. 2015. “Precision‐Engineering the *Pseudomonas aeruginosa* Genome With Two‐Step Allelic Exchange.” Nature Protocols 10, no. 11: 1820–1841. 26492139 10.1038/nprot.2015.115 PMC4862005

[mbt270228-bib-0029] Hopper, D. J. 1976. “The Hydroxylation of *p*‐Cresol and Its Conversion to *p*‐Hydroxybenzaldehyde in *Pseudomonas putida* .” Biochemical and Biophysical Research Communications 69, no. 2: 462–468. 1267796 10.1016/0006-291x(76)90544-1

[mbt270228-bib-0030] Huang, Y. , K.‐X. Zhao , X.‐H. Shen , M. T. Chaudhry , C.‐Y. Jiang , and S.‐J. Liu . 2006. “Genetic Characterization of the Resorcinol Catabolic Pathway in *Corynebacterium glutamicum* .” Applied and Environmental Microbiology 72, no. 11: 7238–7245. 16963551 10.1128/AEM.01494-06 PMC1636210

[mbt270228-bib-0031] Hudlicky, T. , and J. Reed . 2009. “Celebrating 20 Years of SYNLETT – Special Account on the Merits of Biocatalysis and the Impact of Arene *Cis*‐Dihydrodiols on Enantioselective Synthesis.” Synlett 2009: 685–703.

[mbt270228-bib-0032] Jenkins, G. N. , D. W. Ribbons , D. A. Widdowson , A. M. Z. Slawin , and D. J. Williams . 1995. “Synthetic Application of Biotransformations: Absolute Stereochemistry and Diels‐Alder Reactions of the (1*S*,2*R*)‐1,2‐Dihydroxycyclohexa‐3,5‐Diene‐1‐Carboxylic Acid From *Pseudomonas putida* .” Journal of the Chemical Society, Perkin Transactions 81, no. 20: 2647–2655.

[mbt270228-bib-0033] Jerina, D. , J. Daly , B. Witkop , P. Zaltzman‐Nirenberg , and S. Udenfriend . 1968a. “Role of the Arene Oxide‐Oxepin System in the Metabolism of Aromatic Substrates: I. In Vitro Conversion of Benzene Oxide to a Premercapturic Acid and a Dihydrodiol.” Archives of Biochemistry and Biophysics 128, no. 1: 176–183.

[mbt270228-bib-0034] Jerina, D. M. , J. W. Daly , B. Witkop , P. Zaltzman‐Nirenberg , and S. Udenfriend . 1968b. “The Role of Arene Oxide‐Oxepin Systems in the Metabolism of Aromatic Substrates.: III. Formation of 1,2‐Naphthalene Oxide From Naphthalene by Liver Microsomes.” Journal of the American Chemical Society 90, no. 23: 6525–6527. 5682453 10.1021/ja01025a058

[mbt270228-bib-0035] Jiménez, J. I. , B. Miñambres , J. L. García , and E. Díaz . 2002. “Genomic Analysis of the Aromatic Catabolic Pathways From *Pseudomonas putida* KT2440.” Environmental Microbiology 4, no. 12: 824–841. 12534466 10.1046/j.1462-2920.2002.00370.x

[mbt270228-bib-0036] Johnson, R. A. 2004. “Microbial Arene Oxidations.” Organic Reactions 63: 117–264.

[mbt270228-bib-0037] Jung, P. M. J. , W. B. Motherwell , and A. S. Williams . 1997. “Stereochemical Observations on the Bromate Induced Monobromopentahydroxylation of Benzene by Catalytic Photoinduced Charge Transfer Osmylation. A Concise Synthesis of (±)‐Pinitol.” Chemical Communications 14: 1283–1284.

[mbt270228-bib-0038] Kasperek, G. J. , T. C. Bruice , H. Yagi , and D. M. Jerina . 1972. “Differentiation Between the Concerted and Stepwise Mechanisms for Aromatization (NIH‐Shift) of Arene Epoxides.” Journal of the Chemical Society, Chemical Communications 13: 784.

[mbt270228-bib-0039] Kim, S.‐K. , S.‐J. Im , D.‐H. Yeom , and J.‐H. Lee . 2012. “AntR‐Mediated Bidirectional Activation of *antA* and *antR*, Anthranilate Degradative Genes in *Pseudomonas aeruginosa* .” Gene 505, no. 1: 146–152. 22609066 10.1016/j.gene.2012.05.004

[mbt270228-bib-0040] König, G. , M. T. Reetz , and W. Thiel . 2018. “1‐Butanol as a Solvent for Efficient Extraction of Polar Compounds From Aqueous Medium: Theoretical and Practical Aspects.” Journal of Physical Chemistry. B 122, no. 27: 6975–6988. 29897756 10.1021/acs.jpcb.8b02877

[mbt270228-bib-0041] Leveau, J. H. J. , and S. Gerards . 2008. “Discovery of a Bacterial Gene Cluster for Catabolism of the Plant Hormone Indole 3‐Acetic Acid.” FEMS Microbiology Ecology 65, no. 2: 238–250. 18205812 10.1111/j.1574-6941.2008.00436.x

[mbt270228-bib-0042] Marín, M. , I. Plumeier , and D. H. Pieper . 2012. “Degradation of 2,3‐Dihydroxybenzoate by a Novel Meta‐Cleavage Pathway.” Journal of Bacteriology 194, no. 15: 3851–3860. 22609919 10.1128/JB.00430-12 PMC3416551

[mbt270228-bib-0043] Martin, B. , O. Humbert , M. Camara , et al. 1992. “A Highly Conserved Repeated DNA Element Located in the Chromosome of *Streptococcus pneumoniae* .” Nucleic Acids Research 20, no. 13: 3479–3483. 1630918 10.1093/nar/20.13.3479 PMC312505

[mbt270228-bib-0044] Martin, V. J. , and W. W. Mohn . 2000. “Genetic Investigation of the Catabolic Pathway for Degradation of Abietane Diterpenoids by *Pseudomonas abietaniphila* BKME‐9.” Journal of Bacteriology 182, no. 13: 3784–3793. 10850995 10.1128/jb.182.13.3784-3793.2000 PMC94551

[mbt270228-bib-0045] Meier‐Kolthoff, J. P. , J. S. Carbasse , R. L. Peinado‐Olarte , and M. Göker . 2022. “TYGS and LPSN: A Database Tandem for Fast and Reliable Genome‐Based Classification and Nomenclature of Prokaryotes.” Nucleic Acids Research 50, no. D1: D801–D807. 34634793 10.1093/nar/gkab902 PMC8728197

[mbt270228-bib-0046] Meier‐Kolthoff, J. P. , and M. Göker . 2019. “TYGS Is an Automated High‐Throughput Platform for State‐Of‐The‐Art Genome‐Based Taxonomy.” Nature Communications 10, no. 1: 2182. 10.1038/s41467-019-10210-3 PMC6522516 31097708

[mbt270228-bib-0047] Muraki, T. , M. Taki , Y. Hasegawa , H. Iwaki , and P. C. K. Lau . 2003. “Prokaryotic Homologs of the Eukaryotic 3‐Hydroxyanthranilate 3,4‐Dioxygenase and 2‐Amino‐3‐Carboxymuconate‐6‐Semialdehyde Decarboxylase in the 2‐Nitrobenzoate Degradation Pathway of *Pseudomonas fluorescens* Strain KU‐7.” Applied and Environmental Microbiology 69, no. 3: 1564–1572. 12620844 10.1128/AEM.69.3.1564-1572.2003 PMC150085

[mbt270228-bib-0048] Nakatsu, C. H. , N. A. Straus , and R. C. Wyndham . 1995. “The Nucleotide Sequence of the Tn*5271* 3‐Chlorobenzoate 3,4‐Dioxygenase Genes (*cbaAB*) Unites the Class IA Oxygenases in a Single Lineage.” Microbiology 141, no. Pt 2: 485–495. 7704279 10.1099/13500872-141-2-485

[mbt270228-bib-0049] Nogales, J. , A. Canales , J. Jiménez‐Barbero , J. L. García , and E. Díaz . 2005. “Molecular Characterization of the Gallate Dioxygenase From *Pseudomonas putida* KT2440. The Prototype of a New Subgroup of Extradiol Dioxygenases.” Journal of Biological Chemistry 280, no. 42: 35382–35390. 16030014 10.1074/jbc.M502585200

[mbt270228-bib-0050] Ohta, Y. , and D. W. Ribbons . 1976. “Bacterial Metabolism of Resorcinylic Compounds: Purification and Properties of Orcinol Hydroxylase and Resorcinol Hydroxylase From *Pseudomonas putida* ORC.” European Journal of Biochemistry 61, no. 1: 259–269. 1280 10.1111/j.1432-1033.1976.tb10019.x

[mbt270228-bib-0051] Olivera, E. R. , B. Miñambres , B. García , et al. 1998. “Molecular Characterization of the Phenylacetic Acid Catabolic Pathway in *Pseudomonas putida* U: The Phenylacetyl‐CoA Catabolon.” Proceedings of the National Academy of Sciences of the United States of America 95, no. 11: 6419–6424. 9600981 10.1073/pnas.95.11.6419 PMC27761

[mbt270228-bib-0052] Ornston, L. N. , and R. Y. Stanier . 1966. “The Conversion of Catechol and Protocatechuate to β‐Ketoadipate by *Pseudomonas putida*: I. Biochemistry.” Journal of Biological Chemistry 241, no. 16: 3776–3786. 5916391

[mbt270228-bib-0053] Pérez‐Pantoja, D. , B. González , and D. H. Pieper . 2010. “Aerobic Degradation of Aromatic Hydrocarbons.” In Handbook of Hydrocarbon and Lipid Microbiology, edited by K. N. Timmis , 799–837. Springer Berlin Heidelberg.

[mbt270228-bib-0054] Reed, J. W. , and T. Hudlicky . 2015. “The Quest for a Practical Synthesis of Morphine Alkaloids and Their Derivatives by Chemoenzymatic Methods.” Accounts of Chemical Research 48, no. 3: 674–687. 25730681 10.1021/ar500427k

[mbt270228-bib-0055] Reetz, M. T. , and G. König . 2021. “(2021) *n*‐Butanol: An Ecologically and Economically Viable Extraction Solvent for Isolating Polar Products From Aqueous Solutions.” European Journal of Organic Chemistry 46: 6224–6228.

[mbt270228-bib-0056] Reiner, A. M. 1971. “Metabolism of Benzoic Acid by Bacteria: 3,5‐Cyclohexadiene‐1,2‐Diol‐1‐Carboxylic Acid Is an Intermediate in the Formation of Catechol.” Journal of Bacteriology 108, no. 1: 89–94. 4399343 10.1128/jb.108.1.89-94.1971 PMC247036

[mbt270228-bib-0057] Reiner, A. M. , and G. D. Hegeman . 1971. “Metabolism of Benzoic Acid by Bacteria. Accumulation of (−)‐3,5‐Cyclohexadiene‐1,2‐Diol‐1‐Carboxylic Acid by a Mutant Strain of *Alcaligenes eutrophus* .” Biochemistry 10, no. 13: 2530–2536. 4326771 10.1021/bi00789a017

[mbt270228-bib-0058] Ribbons, D. W. 1966. “Bacterial Oxidation of 2,3‐Dihydroxybenzoic Acid—A New Oxygenase.” Biochemical Journal 99, no. 2: 30P–31P.

[mbt270228-bib-0059] Ribbons, D. W. , and P. J. Senior . 1970. “2,3‐Dihydroxybenzoate 3,4‐Oxygenase From *Pseudomonas Fluorescens*: Determination of the Site of Ring Cleavage With a Substrate Analogue.” Biochemical Journal 117, no. 2: 28P–29P. 10.1042/bj1170028p PMC1178909 5420036

[mbt270228-bib-0060] Schäfer, A. , A. Tauch , W. Jäger , J. Kalinowski , G. Thierbach , and A. Pühler . 1994. “Small Mobilizable Multi‐Purpose Cloning Vectors Derived From the *Escherichia coli* Plasmids pK18 and pK19: Selection of Defined Deletions in the Chromosome of *Corynebacterium glutamicum* .” Gene 145, no. 1: 69–73. 8045426 10.1016/0378-1119(94)90324-7

[mbt270228-bib-0061] Shen, W. , W. Liu , J. Zhang , et al. 2010. “Cloning and Characterization of a Gene Cluster Involved in the Catabolism of *p*‐Nitrophenol From *Pseudomonas putida* DLL‐E4.” Bioresource Technology 101, no. 19: 7516–7522. 20466541 10.1016/j.biortech.2010.04.052

[mbt270228-bib-0062] Shingler, V. , J. Powlowski , and U. Marklund . 1992. “Nucleotide Sequence and Functional Analysis of the Complete Phenol/3,4‐Dimethylphenol Catabolic Pathway of *Pseudomonas* sp. Strain CF600.” Journal of Bacteriology 174, no. 3: 711–724. 1732207 10.1128/jb.174.3.711-724.1992 PMC206147

[mbt270228-bib-0063] Southgate, E. H. , J. Pospech , J. Fu , D. R. Holycross , and D. Sarlah . 2016. “Dearomative Dihydroxylation With Arenophiles.” Nature Chemistry 8, no. 10: 922–928. 10.1038/nchem.2594 PMC5971114 27657867

[mbt270228-bib-0064] Studier, F. W. 2005. “Protein Production by Auto‐Induction in High Density Shaking Cultures.” Protein Expression and Purification 41, no. 1: 207–234. 15915565 10.1016/j.pep.2005.01.016

[mbt270228-bib-0065] Suen, W.‐C. 1991. “Gene Expression of Naphthalene Dioxygenase From Pseudomonas sp.” In NCIB 9816–4 in *Escherichia coli* . Iowa City.

[mbt270228-bib-0066] Sun, S.‐Y. , X. Zhang , Q. Zhou , J.‐C. Chen , and G.‐Q. Chen . 2008. “Microbial Production of *Cis*‐1,2‐Dihydroxy‐Cyclohexa‐3,5‐Diene‐1‐Carboxylate by Genetically Modified *Pseudomonas putida* .” Applied Microbiology and Biotechnology 80, no. 6: 977–984. 18679676 10.1007/s00253-008-1603-2

[mbt270228-bib-0067] Tago, K. , J. Sato , H. Takesa , H. Kawagishi , and M. Hayatsu . 2005. “Characterization of Methylhydroquinone‐Metabolizing Oxygenase Genes Encoded on Plasmid in *Burkholderia* sp. NF100.” Journal of Bioscience and Bioengineering 100, no. 5: 517–523. 16384790 10.1263/jbb.100.517

[mbt270228-bib-0068] Taylor, S. J. , D. W. Ribbons , A. M. Slawin , D. A. Widdowson , and D. J. Williams . 1987. “Biochemically Generated Chiral Intermediates for Organic Synthesis: The Absolute Stereochemistry of 4‐Bromo‐*Cis*‐2,3‐Dihydroxycyclohexa‐4,6‐Diene‐1‐Carboxylic Acid Formed From 4‐Bromobenzoic Acid by a Mutant of *Pseudomonas putida* .” Tetrahedron Letters 28, no. 50: 6391–6392.

[mbt270228-bib-0069] Untergasser, A. , I. Cutcutache , T. Koressaar , et al. 2012. “Primer3–New Capabilities and Interfaces.” Nucleic Acids Research 40, no. 15: e115. 22730293 10.1093/nar/gks596 PMC3424584

[mbt270228-bib-0070] Werlen, C. , H. P. Kohler , and J. R. van der Meer . 1996. “The Broad Substrate Chlorobenzene Dioxygenase and *Cis*‐Chlorobenzene Dihydrodiol Dehydrogenase of *Pseudomonas* sp. Strain P51 Are Linked Evolutionarily to the Enzymes for Benzene and Toluene Degradation.” Journal of Biological Chemistry 271, no. 8: 4009–4016. 8626733 10.1074/jbc.271.8.4009

[mbt270228-bib-0071] Wissner, J. L. , J. Ludwig , W. Escobedo‐Hinojosa , and B. Hauer . 2021. “An Enhanced Toluene Dioxygenase Platform for the Production of *Cis*‐1,2‐Dihydrocatechol in *Escherichia coli* BW25113 Lacking Glycerol Dehydrogenase Activity.” Journal of Biotechnology 325: 380–388. 32946884 10.1016/j.jbiotec.2020.09.012

[mbt270228-bib-0072] Worsey, M. J. , and P. A. Williams . 1975. “Metabolism of Toluene and Xylenes by *Pseudomonas putida* (*Arvilla*) mt‐2: Evidence for a New Function of the TOL Plasmid.” Journal of Bacteriology 124, no. 1: 7–13. 1176436 10.1128/jb.124.1.7-13.1975 PMC235858

[mbt270228-bib-0073] Yildirim, S. , R. Ruinatscha , R. Gross , et al. 2006. “Selective Hydrolysis of the Nitrile Group of *Cis*‐Dihydrodiols From Aromatic Nitriles.” Journal of Molecular Catalysis B: Enzymatic 38, no. 2: 76–83.

[mbt270228-bib-0074] Young, I. , L. Jackman , and F. Gibson . 1969. “The Isolation, Identification and Properties of 2,3‐Dihydro‐2,3‐Dihydroxybenzoic Acid. An Intermediate in the Biosynthesis of 2,3‐Dihydroxybenzoic Acid.” Biochimica et Biophysica Acta 177, no. 3: 381–388. 5787237 10.1016/0304-4165(69)90300-6

[mbt270228-bib-0075] Young, I. G. , L. Langman , R. K. Luke , and F. Gibson . 1971. “Biosynthesis of the Iron‐Transport Compound Enterochelin: Mutants of *Escherichia coli* Unable to Synthesize 2,3‐Dihydroxybenzoate.” Journal of Bacteriology 106, no. 1: 51–57. 4928016 10.1128/jb.106.1.51-57.1971 PMC248643

