# Supplementary material for: Biotechnological Production, Isolation and Characterisation of (2R ,3S )‐2,3‐Dihydroxy‐2,3‐Dihydrobenzoate
Source: Microb Biotechnol. 2025 Sep 17;18(9):e70228. doi: 10.1111/1751-7915.70228 (PMC12442788; doi:10.1111/1751-7915.70228)
Supplement: Supplementary file 1 — Data S1: mbt270228‐sup‐0001‐Supinfo.docx. [file MBT2-18-e70228-s001.docx]

**Biotechnological Production, Isolation, and Characterization of (2*R*,3*S*)-2,3-Dihydroxy-2,3-dihydrobenzoate**

Martina Kiel^1^; Israel Barrantes^2^; Dietmar H. Pieper^2^; Karl-Heinrich Engesser^1^

^1^ Institute of Sanitary Engineering, Water Quality and Solid Waste Management, University of Stuttgart, D-70569 Stuttgart, Germany

^2^ *Microbial Interactions and Processes Research Group,* Helmholtz Centre for Infection Research, D-38124 Braunschweig, Germany

**Supporting Information**

**Supplementary Figures**


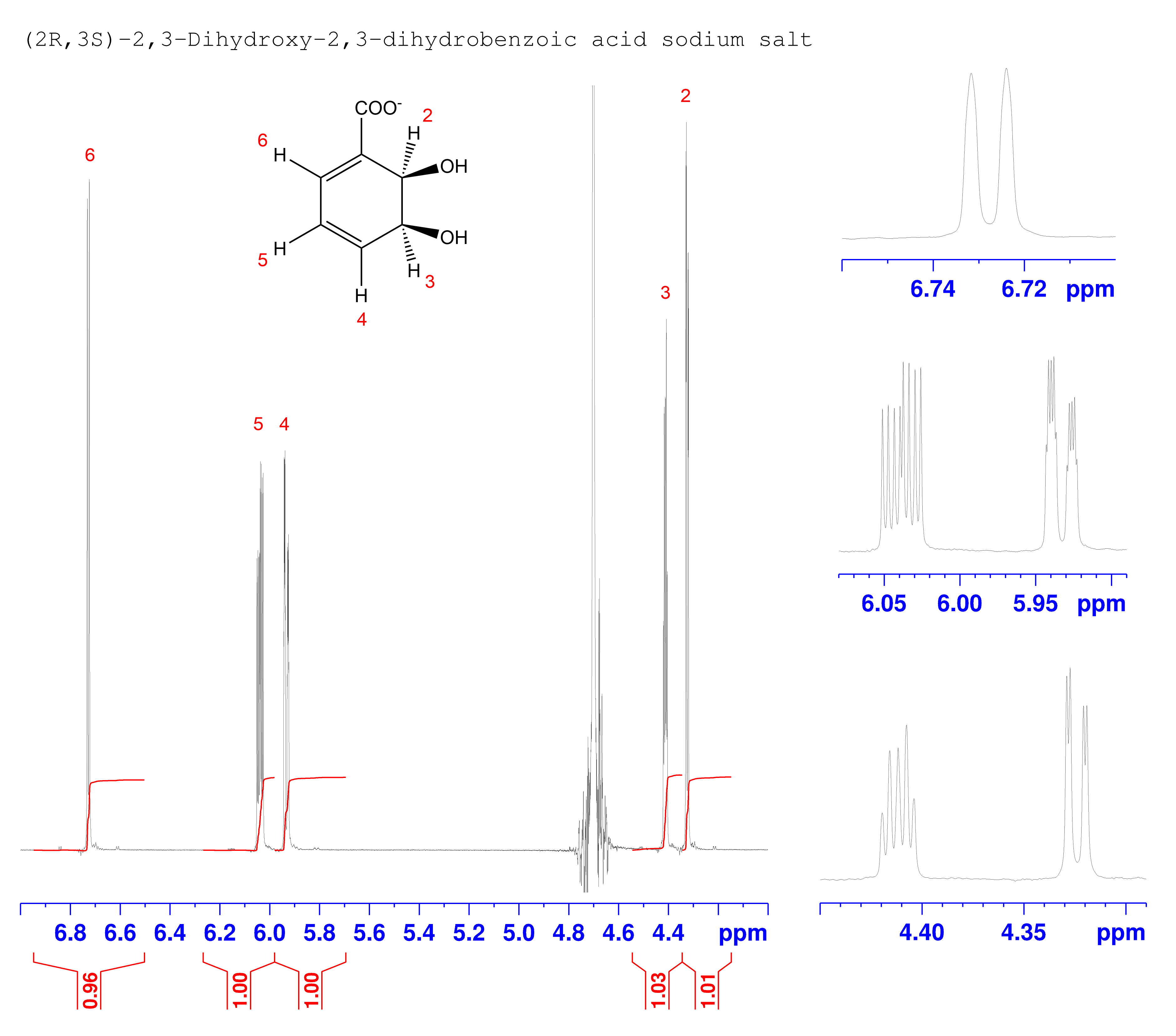


**Fig. S1**: ^1^H-NMR spectrum of *cis*-2,3-DD sodium salt (monohydrate). The peak at 4.7 ppm is caused by H_2_O hydrogen.


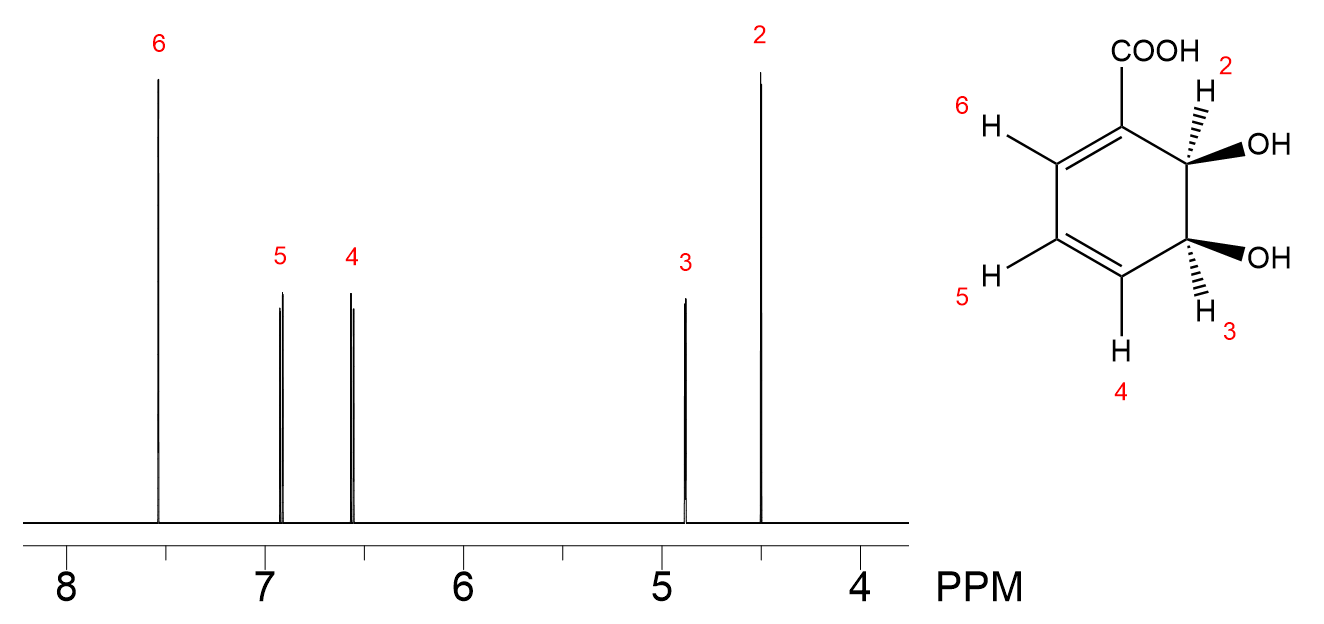


**Fig. S2**: Simulated ^1^H-NMR spectrum of 2,3-DD at 800 MHz. The spectrum was generated using the NMR predictor available at https://www.nmrdb.org (Castillo *et al.*, 2011).


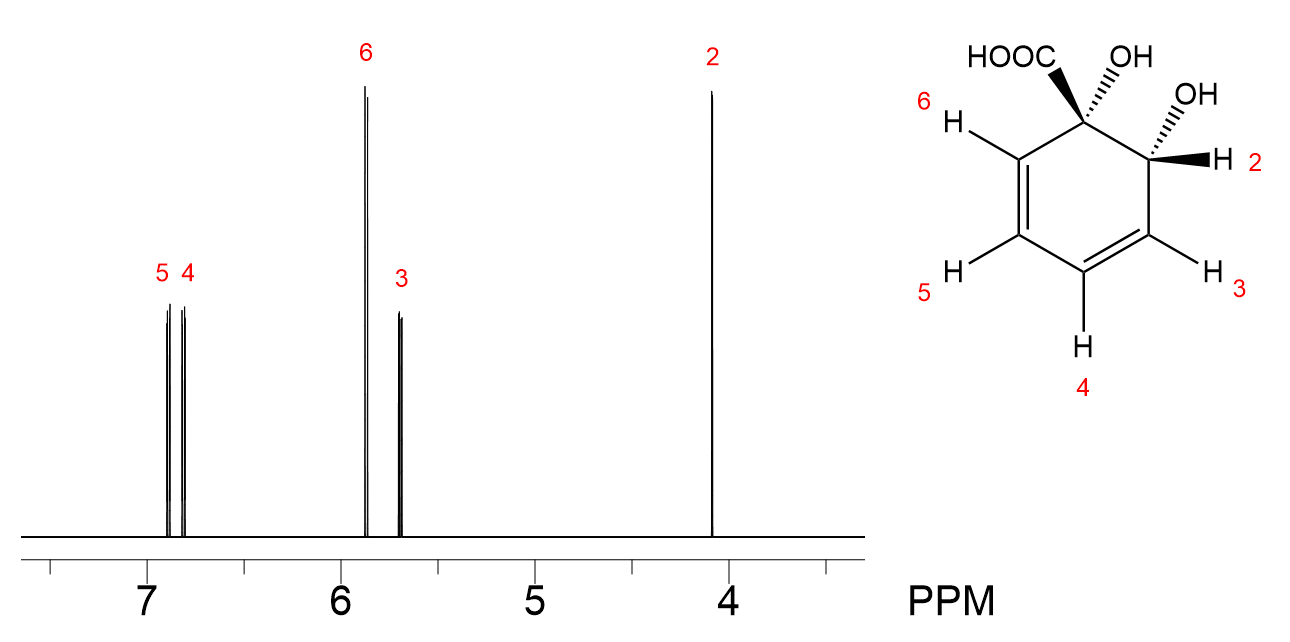


**Fig. S3**: Simulated ^1^H-NMR spectrum of 1,2-DD at 800 MHz. The spectrum was generated using the NMR predictor available at https://www.nmrdb.org (Castillo *et al.*, 2011).


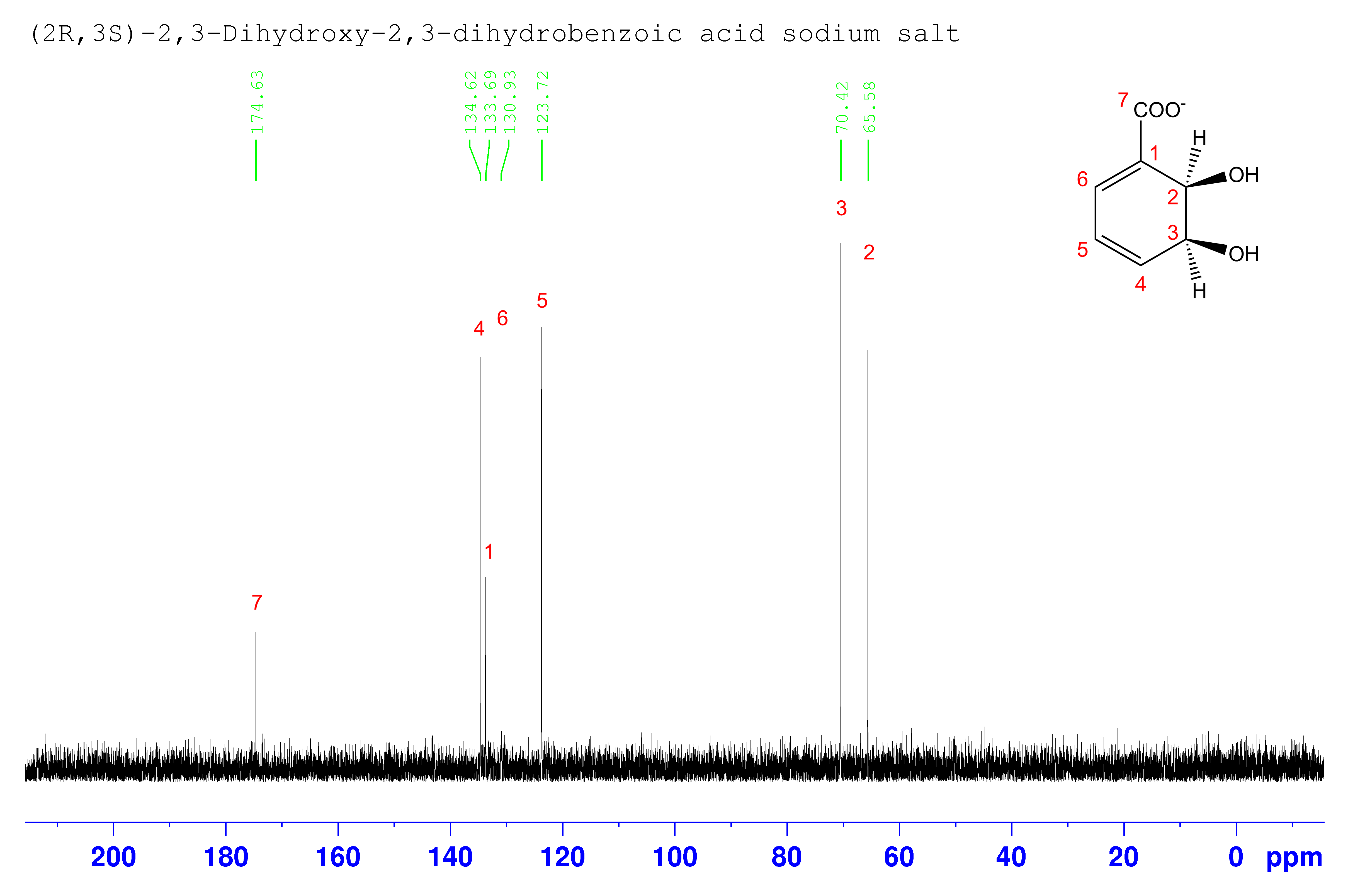


**Fig. S4**: ^13^C-NMR spectrum of *cis*-2,3-DD sodium salt (monohydrate)


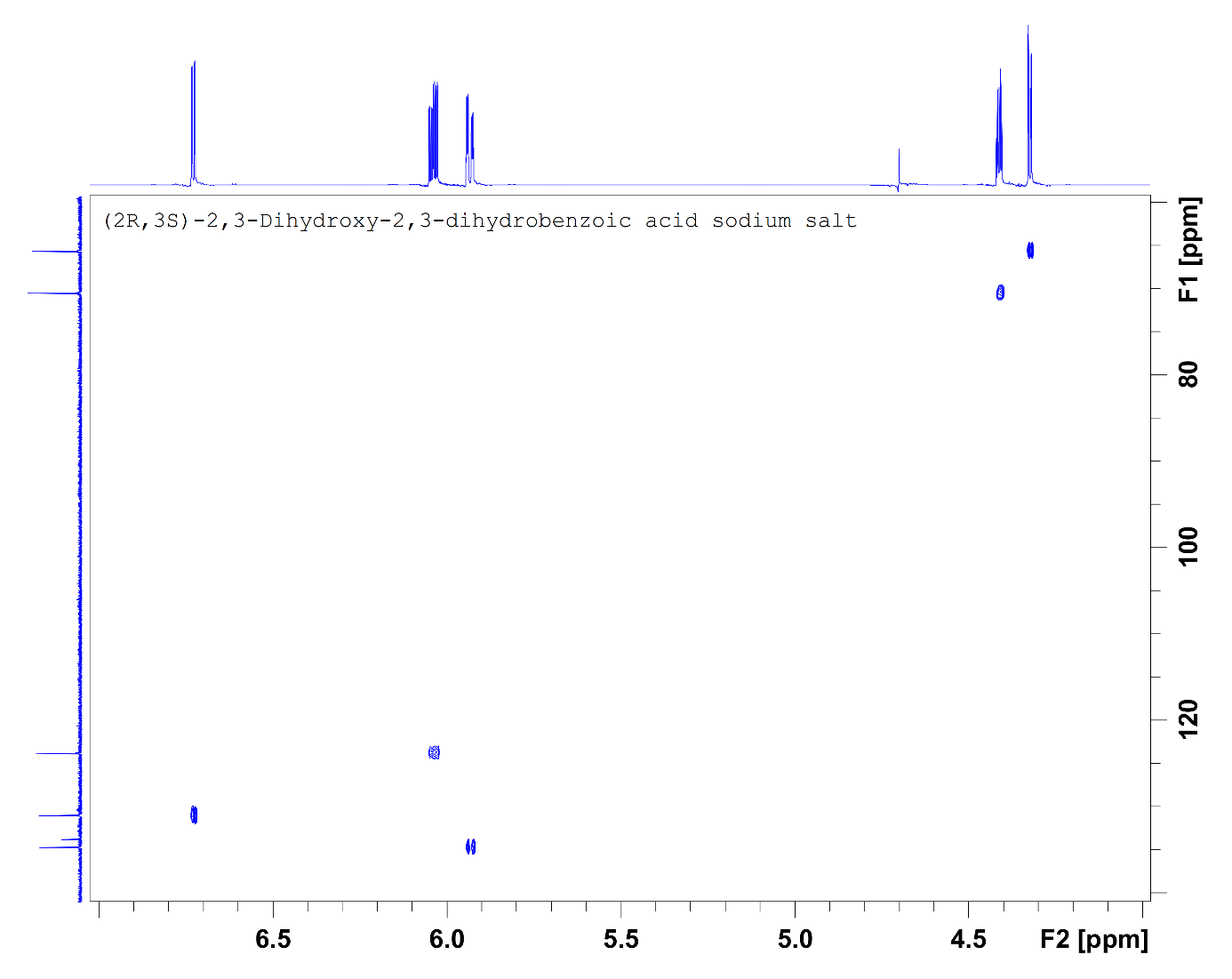


**Fig. S5**: HSQC spectrum of *cis*-2,3-DD sodium salt (monohydrate). The ^13^C peak at 174.63 ppm representing the carboxyl C is omitted.


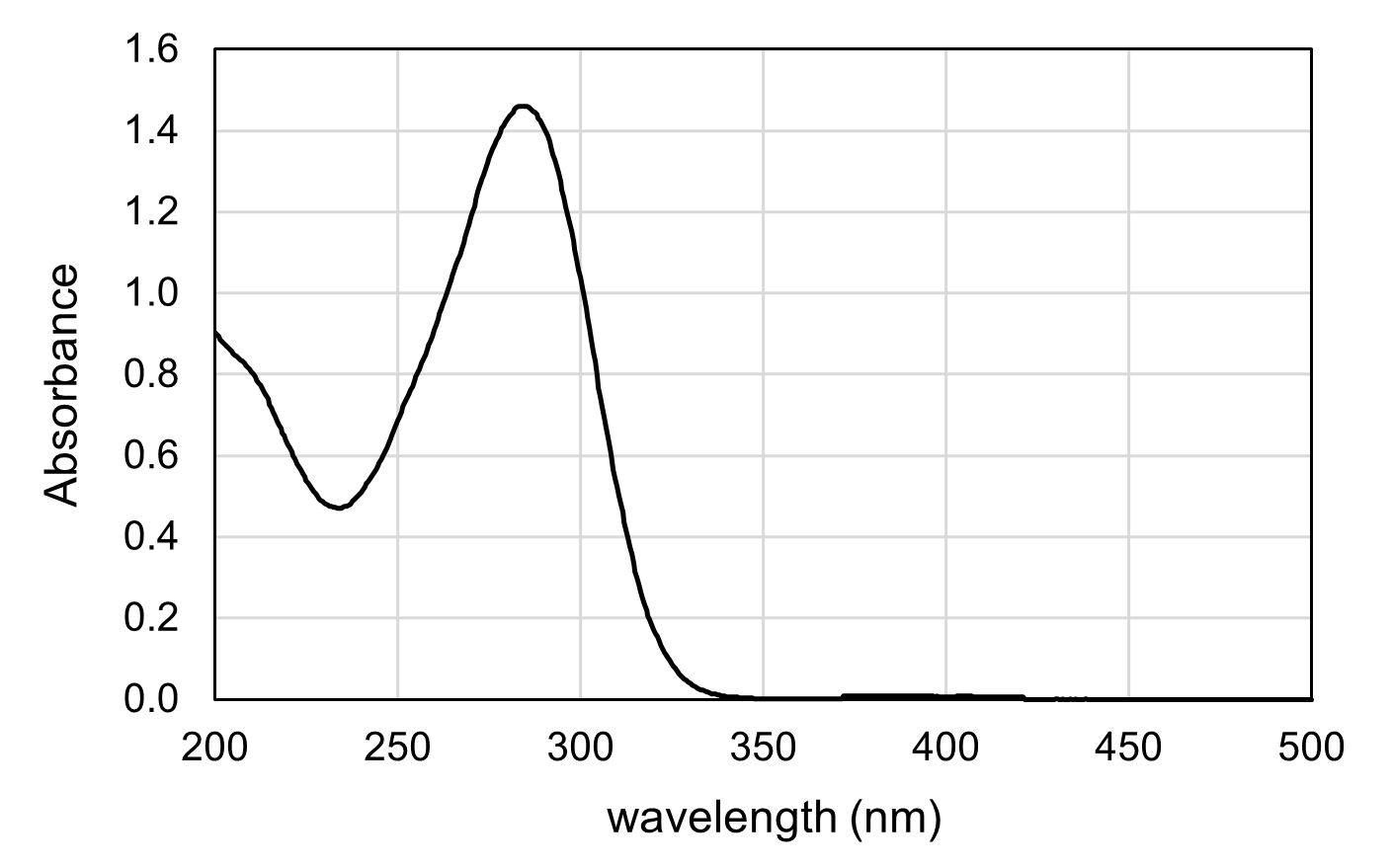


**Fig. S6**: UV/Vis spectrum of *cis*-2,3-DD sodium salt (0.25 mM in H_2_O). Data were obtained at room temperature using a Heλios γ spectrophotometer (Thermo Scientific).


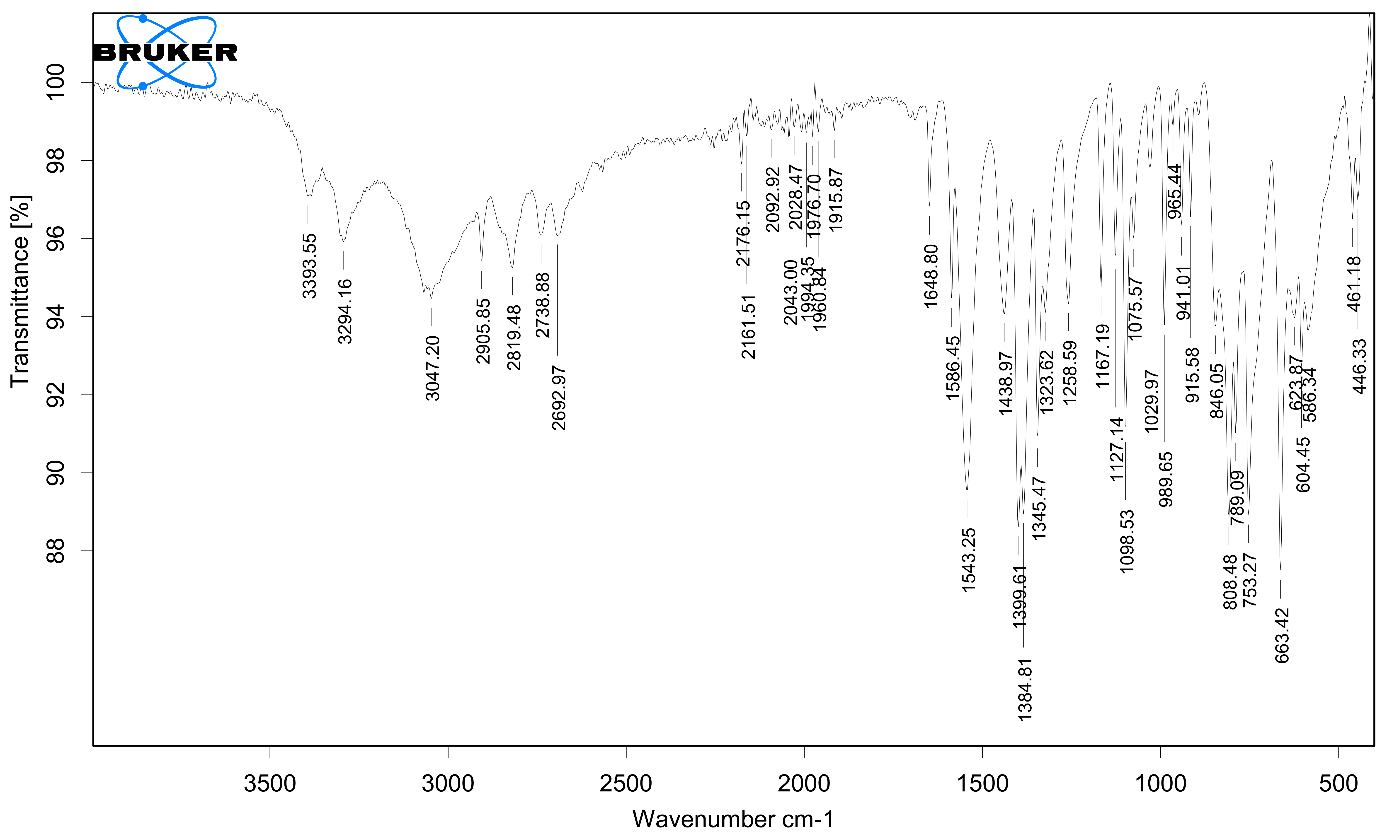


**Fig. S7**: IR absorption of *cis*-2,3-DD sodium salt (monohydrate).


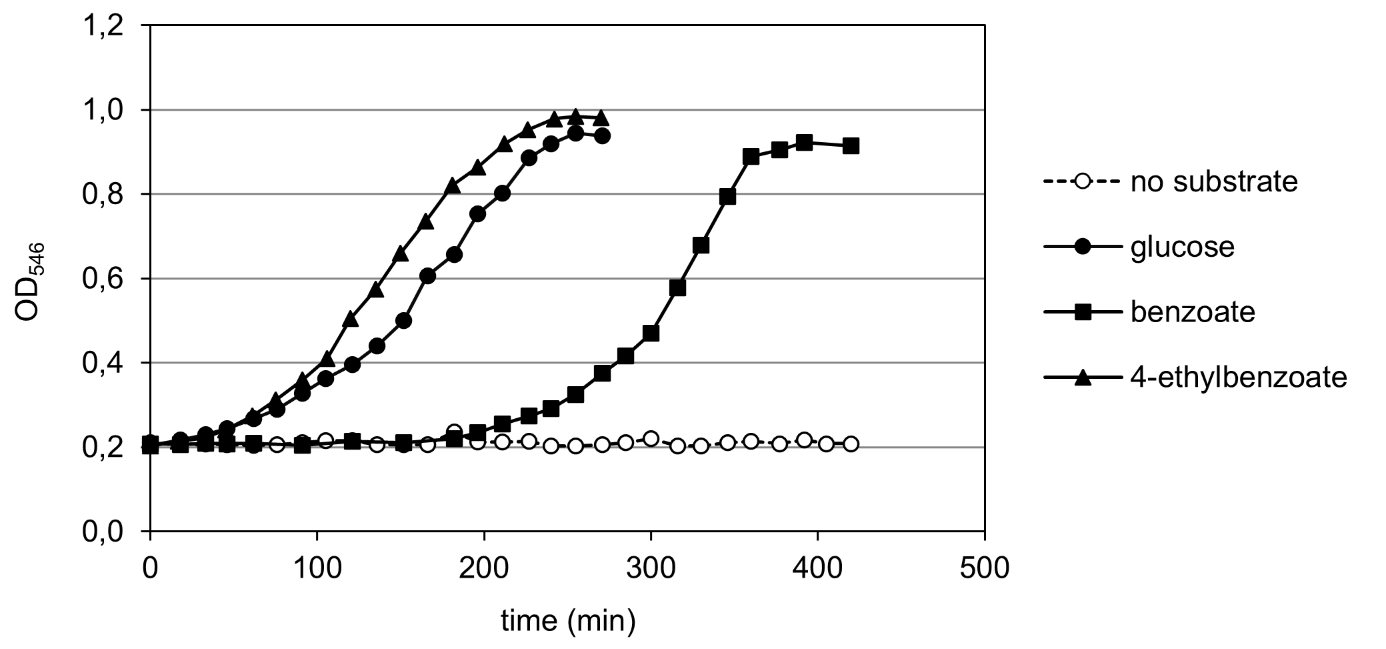


**Fig. S8**: Induction experiments with glucose-grown EB200 (wild type) (n=1). Substrates were added at a concentration of 3 mM.


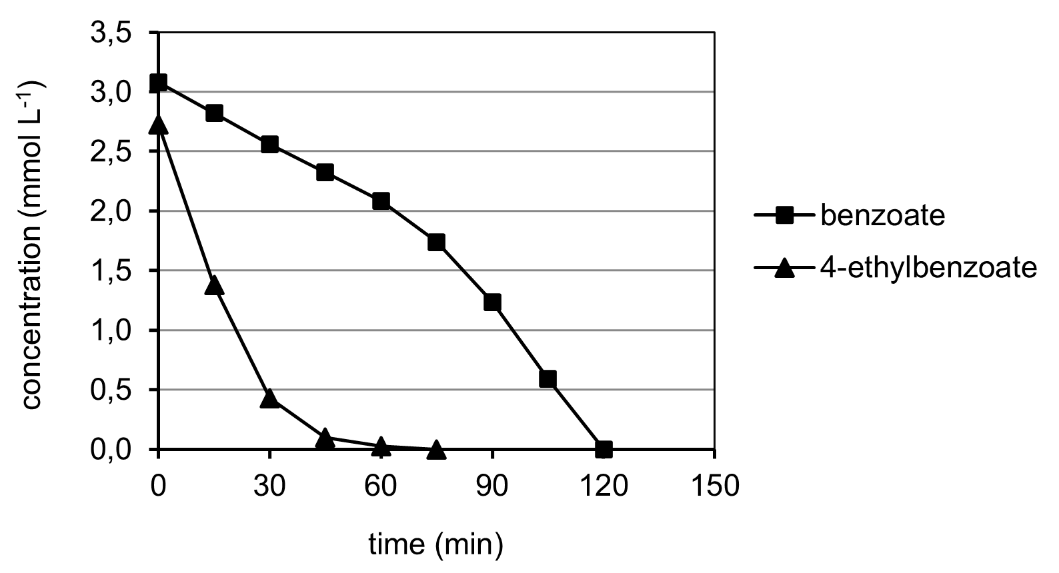


**Fig. S9**: Transformation of benzoates by glucose-grown EB200 (wild type) in mineral media at OD_546_ = 4.5 as the start value (n=1).

**
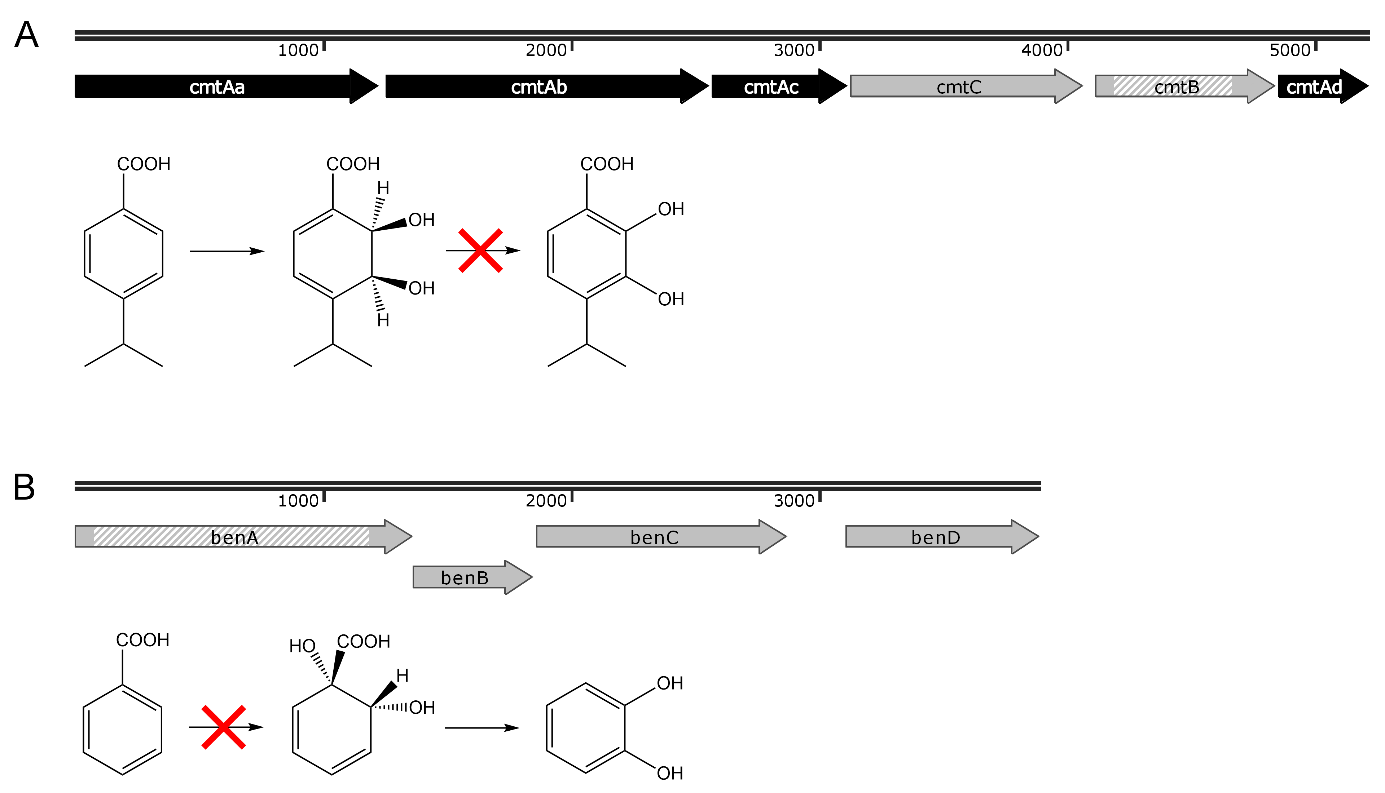
**

**Fig. S10:** Partial *p*-cumate (A) and benzoate (B) degradative gene clusters in EB200. Black arrows indicate genes encoding the four components of *p*-cumate 2,3-dioxygenase CmtA; shaded areas qualitatively indicate deleted regions in EB200 ∆*cmtB* or ∆*benA*, respectively. Schemes depict the reaction steps blocked in the mutants. *cmtAa*, PCDO ferredoxin reductase; *cmtAb*, PCDO large subunit; *cmtAc*, PCDO small subunit; *cmtC*, 2,3-dihydroxy-*p*-cumate 3,4-dioxygenase; *cmtB*, 2,3-dihydroxy-2,3-dihydro-*p*-cumate dehydrogenase; *cmtAd*, PCDO ferredoxin. *benA*, benzoate 1,2-dioxygenase large subunit; *benB*, benzoate 1,2-dioxygenase small subunit; *benC*, benzoate 1,2-dioxygenase reductase; *benD*, 1,2-dihydroxy-2-hydrobenzoate dehydrogenase. For the respective NCBI Genbank locus tags cf. Table S8.


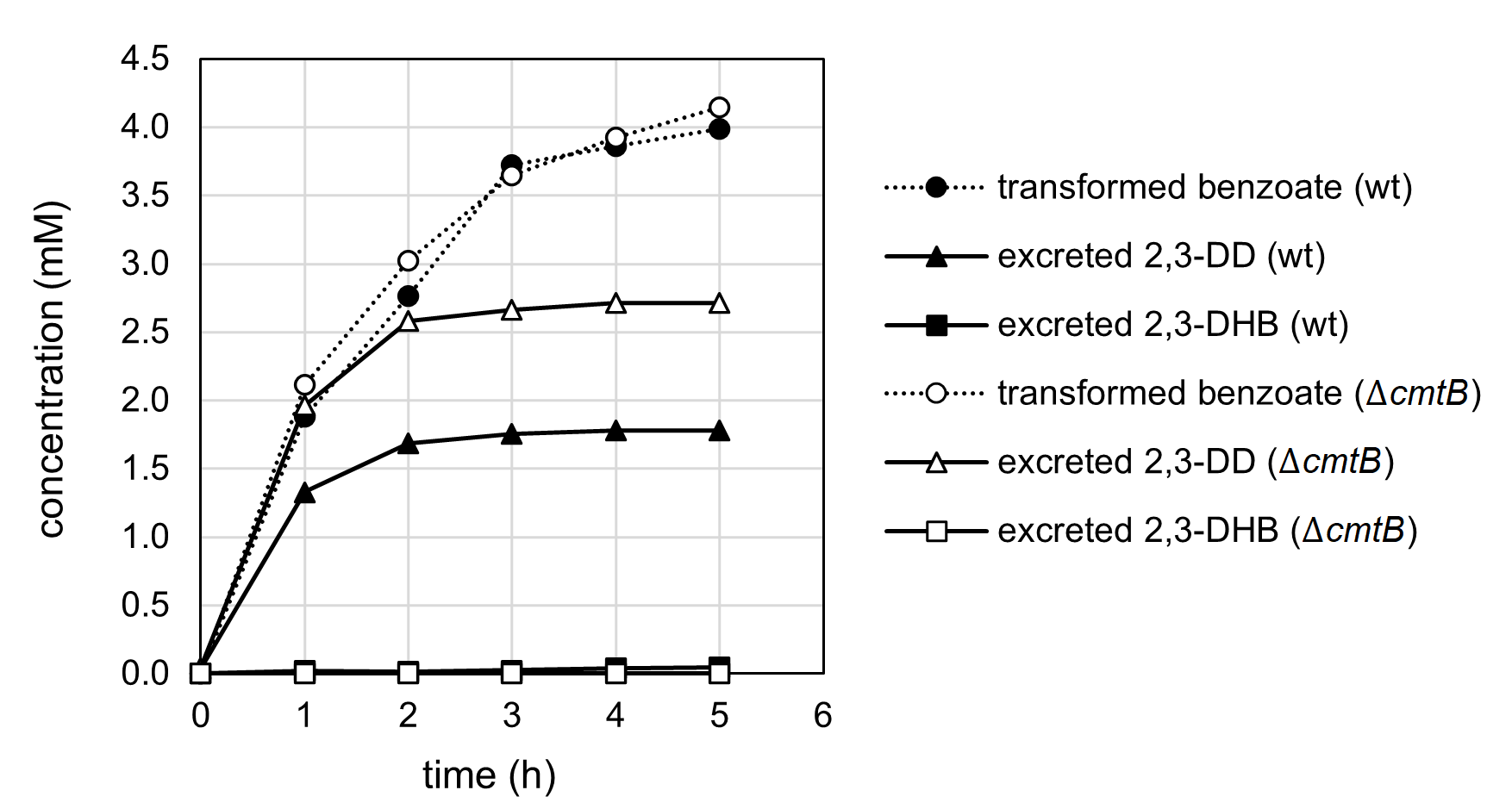


**Fig. S11**: Transformation of benzoate and excretion of 2,3-DD and 2,3-DHB by glucose-grown resting cells of the EB200 wild type (filled symbols, OD_546_ = 8.7) and EB200 ∆*cmtB* mutant (empty symbols, OD_546_ = 8.8) (n=1). Benzoate was added at a concentration of 5 mM at the beginning of the experiment and another 5 mM of benzoate was added at t = 2 h.


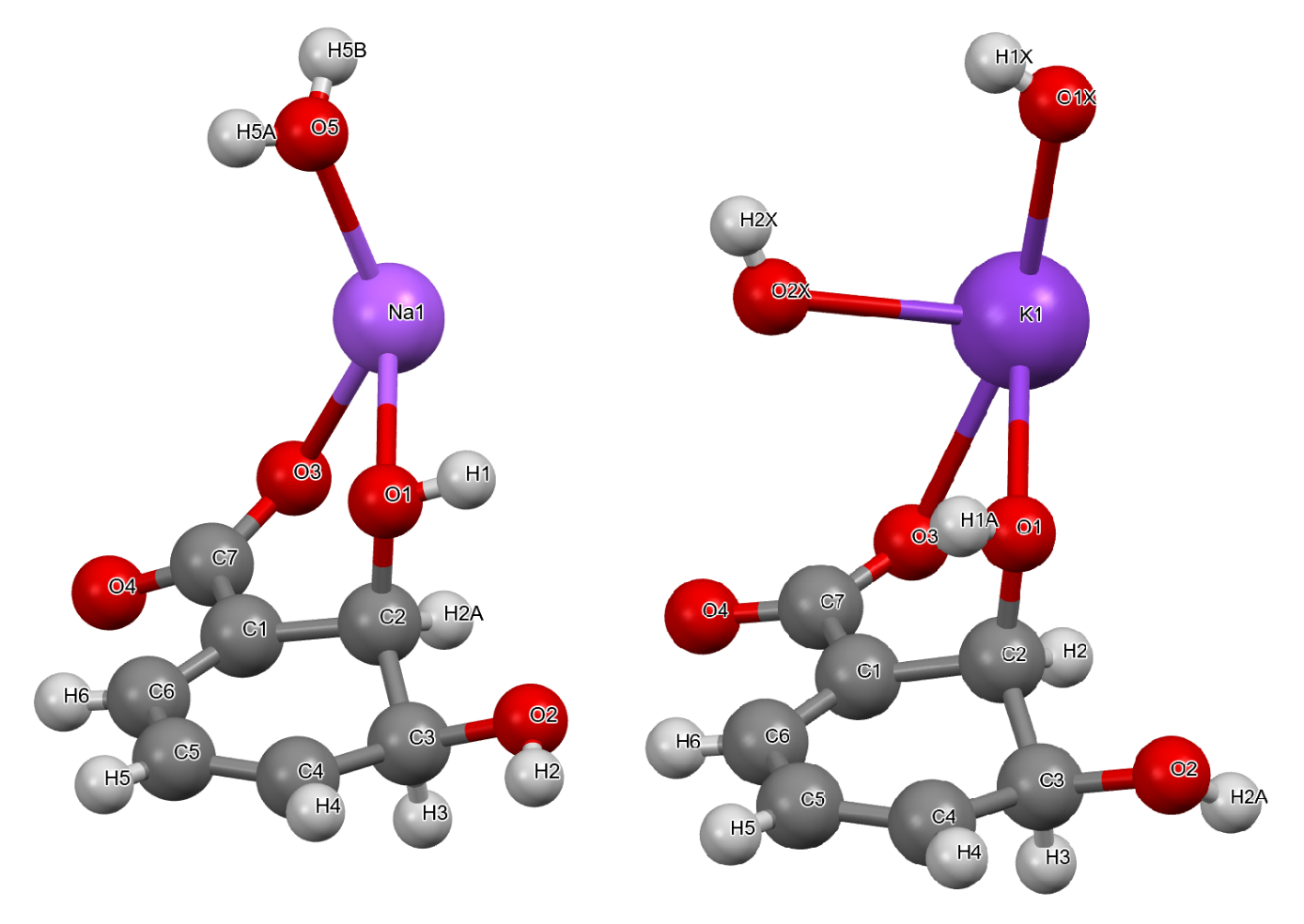


**Fig. S12**: Molecular structures of *cis*-2,3-DD sodium salt monohydrate (left side) and *cis*-2,3-DD potassium salt monohydrate (right side).


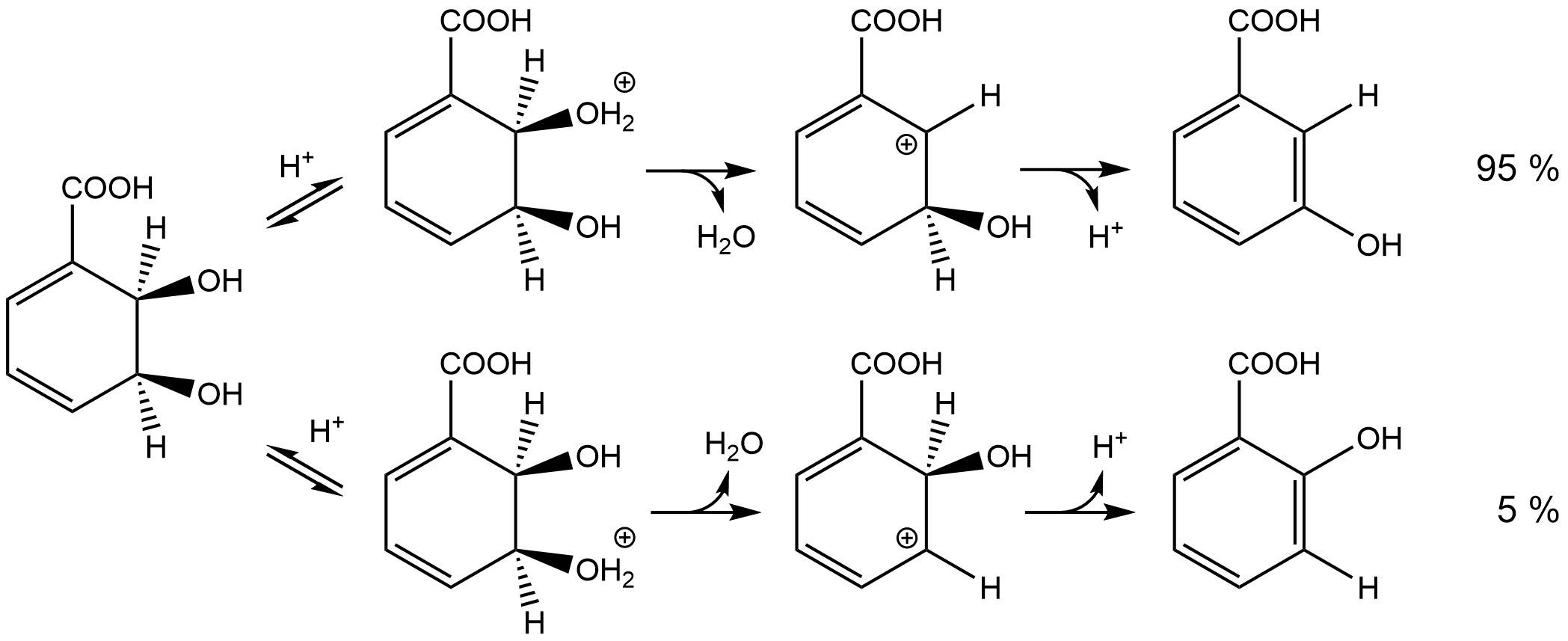


**Fig. S13**: Proposed mechanism of the acid-catalyzed aromatization of 2,3-DD. Due to the electron withdrawing effect of the carboxyl substituent, the *meta*-hydroxylated carbocation (which does not have a resonance structure with a positive charge next to the COOH group) is favored over the *ortho*-hydroxylated isomer (Boyd *et al.*, 1994).

**
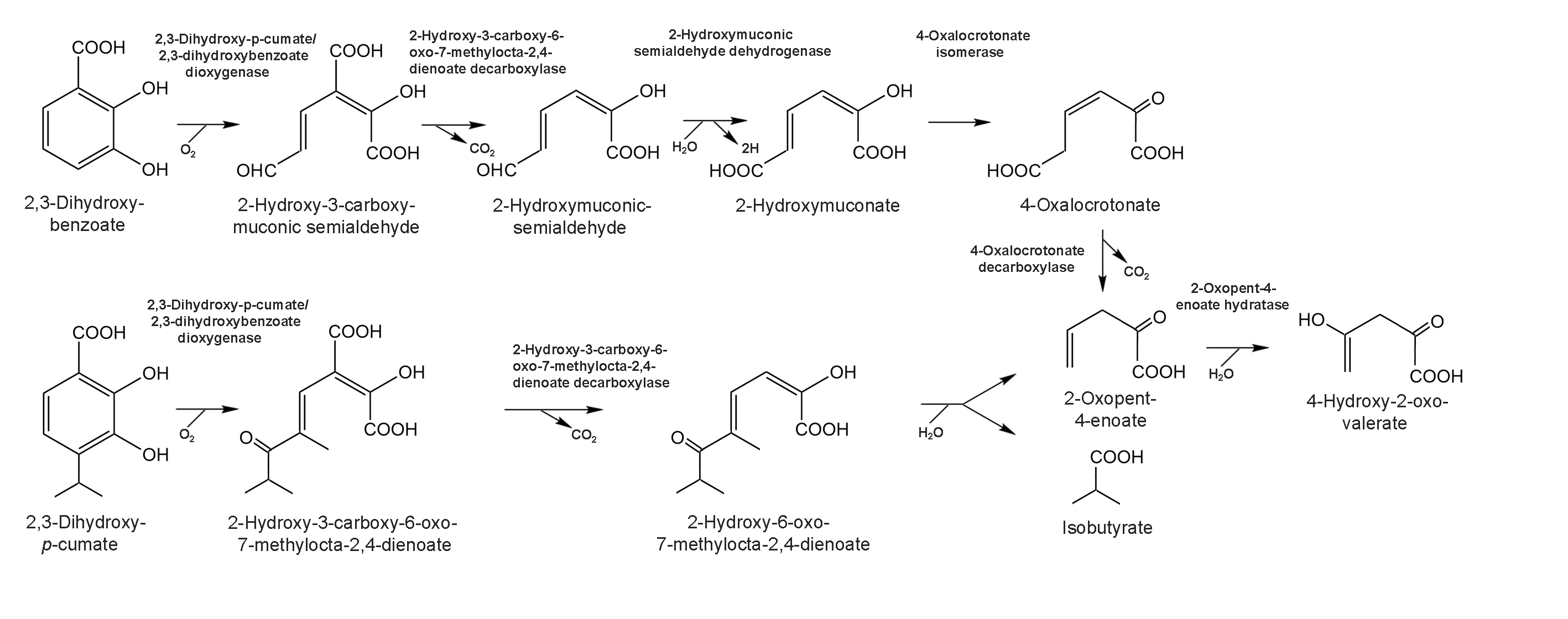
**

**Fig. S14:** Proposed metabolism of 2,3-dihydroxybenzoate and 2,3-dihydroxy-*p*-cumate by the p-cumate/2,3-dihydroxybenzoate pathways

**Supplementary Tables**

**Table S1:** Strains used in this study

| **Strain** | **Characteristics** | **Genbank accessions** | **Reference** |
| --- | --- | --- | --- |
| EB200 | *Pseudomonas citronellolis*  4-ethylbenzoate degrading isolate  positive screening result for 2,3-DD | 16S rDNA: PP921341  WGS data: JBAISM01 | This study |
| EB600 | Neobacillus sp.  4-ethylbenzoate degrading isolate  positive screening result for 2,3-DD | 16S rDNA: PP921342 | This study |
| EB800 | Pseudomonas aeruginosa  4-ethylbenzoate degrading isolate  positive screening result for 2,3-DD | 16S rDNA: PP921343 | This study |
| IB300 | Burkholderia contaminans  p-cumate degrading isolate  positive screening result for 2,3-DD | 16S rDNA: PP921345 | This study |
| IB400 | Pseudomonas citronellolis  p-cumate degrading isolate  positive screening result for 2,3-DD | 16S rDNA: PP921346 | This study |
| IB600 | Mycobacterium mucogenicum  p-cumate degrading isolate  positive screening result for 2,3-DD | 16S rDNA: PP921347 | This study |
| IB800 | Mycobacterium virginiense  p-cumate degrading isolate  positive screening result for 2,3-DD | 16S rDNA: PP921348 | This study |
| ETB I | Pseudomonas monteilii  4-ethylbenzoate degrading strain  positive screening result for 2,3-DD | 16S rDNA: PP921344 | Strain collection of ALR department at ISWA, University of Stuttgart |
| F1 | *Pseudomonas putida*  p-cumate degrading strain  positive screening result for 2,3-DD |  | Eaton, 1996 |
| EB200 ∆*cmtB* | Knock-out mutant of EB200 with a 0.7 kb deletion in the 2,3-dihydroxy-2,3-dihydro-p-cumate dehydrogenase gene cmtB |  | This study |
| EB200 ∆*benA* | Knock-out mutant of EB200 with a 1.3 kb deletion in the benzoate 1,2-dioxygenase large subunit gene benA |  | This study |
| EB200 ∆*cmtB* ∆*benA* | Double knock-out mutant of EB200 lacking both cmtB and benA |  | This study |
| *Escherichia coli* JM109 | end*A1,* rec*A1,* gyr*A96,* thi, hsd*R17* (r_k_^–^, m_k_^+^), rel*A1,* sup*E44*, Δ(lac-pro*AB*), [F´ tra*D36*, pro*AB*, lac*I^q^ZΔM15*]. |  | Yanisch-Perron et al., 1985 |
| *Escherichia coli* BL21(DE3) | F^–^*ompT* *hsdS_B_* (r_B_^–^, m_B_^–^) *gal dcm* (DE3) |  | Studier and Moffatt, 1986 |
| *Escherichia coli* Rosetta™ 2(DE3) | F^–^*ompT hsdS*_B_(r_B_^–^ m_B_^–^) *gal dcm* (DE3) pRARE2 (Cam^R^)  Plasmid pRARE2 contains seven rare-codon tRNA genes |  | Merck-Novagen |
| *Escherichia coli* S17.1 λpir | Tp^r^ Sm^r^ recA thi pro hsdR^−^ M^+^ RP4::2-Tc::Mu-Km::Tn7 λpir |  | Simon *et al.*, 1983 |
| *Escherichia coli* DH5α λpir | *endA1* *hsdR17* *glnV44* (=*supE44*) *thi-1 recA1 gyrA96 relA1* *ϕ80dlacΔ(lacZ)M15 Δ(lacZYA-argF)U169 zdg-232*::Tn*10* *uidA::pir^+^* |  | Platt *et al.*, 2000 |

**Table S2:** Primers used in this study. Underlined letters, restriction sites; bold letters, overlapping sequences.

| **Primer** | **Sequence (5‘ 🡪 3‘)** | **Description** |
| --- | --- | --- |
| Cloning of the strain EB200 CmtA gene: | | |
| EB200_cmtAa_1F | AAAAAGAATTCCTGTTTGTATTTTGAGTGGCGAT | Construction of plasmids pT7-5(EB200_cmtA) (outer primer set 1) and pUC118(EB200_cmtA) (outer primer set 2) |
| EB200_cmtAa_2F | AAAAAAAGCTTCTGTTTGTATTTTGAGTGGCGAT |  |
| EB200_cmtAc_R | **TCATCTCACTGCCCTCACAG**GGTGCTCTCCGTTACAGAATG |  |
| EB200_cmtAd_F | **CTGTGAGGGCAGTGAGATGA** |  |
| EB200_cmtAd_1R | AAAAAAAGCTTCGACGCTCAAGAACGGAAAT |  |
| EB200_cmtAd_2R | AAAAAGAATTCCGACGCTCAAGAACGGAAAT |  |
| EB200_S2F | TGACCATGGGTACGGACAAT | Sequencing primers |
| EB200_S1R | ATTCCACTGGCGATGAAGTC |  |
| Cloning of the strain F1 CmtA gene: | | |
| F1_cmtAa_2F | AAAAAAAGCTTCGTGGGTGCGATACAAGC | Construction of plasmid pUC118(F1_cmtA) |
| F1_cmtAc_R | **TGACGCTGATTACCTGACCG**CGTTGTGCTCCGTTACAGAA |  |
| F1_cmtAd_F | **CGGTCAGGTAATCAGCGTCA** |  |
| F1_cmtAd_2R | AAAAACAATTGGCGCTGTTCAAGAGTGGAAA |  |
| F1_S2F | GCCACACATCCGAGTTCAAA | Sequencing primers |
| F1_S1R | CCGCCAGTAATGAAGTCAGC |  |
| Knock-out of *cmtB* and *benA* in strain EB200: | | |
| EB200_cmtB_up_F | AAAAAGAATTCTGGGAATATGCACCTCAGCC | Amplification of fragments flanking the sequence to be deleted in *cmtB* |
| EB200_cmtB_up_R | **GACCACCGCGACCTGTC** |  |
| EB200_cmtB_down_F | **GACAGGTCGCGGTGGTC**AGCGCAATGCTGTGAGGG |  |
| EB200_cmtB_down_R | AAAAAGGATCCGTAGACAGCGCCATCACATG |  |
| EB200_Del-cmtB_R | caacagtcaccgcaccattg | Verification of knock-out of *cmtB* |
| EB200_Del-cmtB_F | agcatttcgatcggattggc |  |
| EB200_benA_up_F | AAAAAGAATTCcgaagatgagcctgcgc | Amplification of fragments flanking the sequence to be deleted in *benA* |
| EB200_benA_up_R | **gtcgatacccagggtcatgg** |  |
| EB200_benA_down_F | **ccatgaccctgggtatcgac**ctcgaggaggccgtgtga |  |
| EB200_benA_down_R | AAAAAGGATCCggatgttcacgccctgg |  |
| EB200_Del-benA_R | CAGATCGTCGCCAGCAAG | Verification of knock-out of *benA* |
| EB200_Del-benA_F | GGCTTCCTCCTCGCTCAG |  |
| Sequencing primers for plasmid inserts: | | |
| pT7-7_F | GCTGCATGTGTCAGAGGTTT | Sequencing of pT7-5 |
| pT7-7_R | TGCCTCACTGATTAAGCATTGG |  |
| pUCmcsF | gtaacgccagggttttcc | Sequencing of pUC118 |
| pUCmcsR | cttccggctcgtatgttg |  |
| pK19-F | tgcttccggctcgtatg | Sequencing of pK19mobsacB |
| pK19-R | tgtgctgcaaggcgatt |  |
| Identification and differentiation of strains: | | |
| BOXA1R | CTACGGCAAGGCGACGCTGACG | BOX PCR fingerprinting |
| 27F | AGAGTTTGATCMTGGCTCAG | Amplification and sequencing of 16S rDNA |
| 1492R | TACGGYTACCTTGTTACGACTT |  |

**Table S3**: Plasmids used and constructed in this study

| **Plasmid** | **Characteristics** | **Reference** |
| --- | --- | --- |
| pK19mobsacB | Kan^R^  pK19 derivative containing the RP4 mob region and the SacB gene | Schäfer et al., 1994 |
| pDTG141 | Amp^R^  Expression plasmid for NDO from Pseudomonas sp. NCIB 9816-4, vector backbone of plasmid pT7-5 | Suen, 1991 |
| pIP107D | Amp^R^  Expression plasmid for cumene dioxygenase from P. fluorescens IP01, vector backbone of plasmid pUC118 | Aoki et al., 1996 |
| pK19mobsacB(benA) | Kan^R^  pK19mobsacB containing fused flanking sequences of benA | This study |
| pK19mobsacB(cmtB) | Kan^R^  pK19mobsacB containing fused flanking sequences of cmtB | This study |
| pT7-5(EB200_cmtA) | Amp^R^  Expression plasmid for PCDO from *P. citronellolis* EB200 | This study |
| pUC118(EB200_cmtA) | Amp^R^  Expression plasmid for PCDO from *P. citronellolis* EB200 | This study |
| pUC118(F1_cmtA) | Amp^R^  Expression plasmid for PCDO from *P. putida* F1 | This study |

**Table S4**: Aromatic degradative potential of *Pseudomonas citronellolis* EB200

| Aromatic degradation genes and proteins in *P. citronellolis* EB200 | | | |  | Similar proteins of validated pathways | | |
| --- | --- | --- | --- | --- | --- | --- | --- |
| gene  V5264_ | protein | coding strand | annotation | gene acronym | % identity | similar protein | host |
| 03735 | MEG7358774 | - | gallate permease |  |  |  |  |
| 03740 | MEG7358775 | - | gallate dioxygenase |  | 92,3 | AAN68130 | *P. putida* KT2440 |
| 03745 | MEG7358776 | - | OprD porin |  | 81,2 | AAN68129 | *P. putida* KT2440 |
| 07855 | MEG7359584 | - | 4-hydroxy-2-oxovalerate aldolase | CmtG | 94 | AAB62295 | *P. putida* F1 |
| 07860 | MEG7359586 | - | acetaldehyde dehydrogenase (acetylating)" | CmtH | 93 | AAB62294 | *P. putida* F1 |
| 07865 | MEG7359587 | - | 2-hydroxypenta-2,4-dienoate hydratase | CmtF | 85,9 | AAB62293 | *P. putida* F1 |
| 07870 | MEG7359588 | - | 2-hydroxy-6-oxo-7-methylocta-2,4-dienoate hydrolase | CmtE | 89,9 | AAB62292 | *P. putida* F1 |
| 07875 | MEG7359589 | - | tetR family transcriptional regulator |  |  |  |  |
| 07880 | MEG7359590 | - | 2-hydroxy-3-carboxy-6-oxo-7-methylocta-2,4-dienoate decarboxylase | CmtD | 81,3 | AAB62290 | *P. putida* F1 |
| 07885 | MEG7359591 | - | *p*-cumate dioxygenase ferredoxin | CmtAd | 87,3 | AAB62289 | *P. putida* F1 |
| 07890 | MEG7359592 | - | 2,3-dihydro-2,3-dihydroxy-*p*-cumate dehydrogenase | CmtB | 87,1 | AAB62288 | *P. putida* F1 |
| 07895 | MEG7359593 | - | 2,3-dihydroxy-*p*-cumate dioxygenase | CmtD | 90,7 | AAB62287 | *P. putida* F1 |
| 07900 | MEG7359594 | - | *p*-cumate dioxygenase beta subunit | CmtAc | 91,2 | AAB62286 | *P. putida* F1 |
| 07905 | MEG7359595 | - | *p*-cumate dioxygenase alpha subunit | CmtAb | 90,1 | AAB62285 | *P. putida* F1 |
| 07910 | MEG7359596 | - | *p*-cumate dioxygenase ferredoxin reductase | CmtAa | 83,3 | AAB62284 | *P. putida* F1 |
| 07915 | MEG7359597 | - | acetyl-coenzyme A synthetase | CymE | 85,8 | AAB62302 | *P. putida* F1 |
| 07920 | MEG7359598 | - | outer membrane protein | CymD | 82,2 | AAB62301 | *P. putida* F1 |
| 07925 | MEG7359599 | - | *p*-cymene methyl hydroxylase reductase component | CymAb | 88,5 | AAB62300 | *P. putida* F1 |
| 07930 | MEG7359600 | - | hypothetical protein |  |  |  |  |
| 07935 | MEG7359601 | - | *p*-cymene methyl hydroxylase hydroxylase subunit | CymAa | 89,4 | AAB62299 | *P. putida* F1 |
| 07940 | MEG7359602 | - | *p*-cumic aldehyde dehydrogenase | CymC | 92,7 | AAB62298 | *P. putida* F1 |
| 07945 | MEG7359603 | - | *p*-cumic alcohol dehydrogenase | CymB | 95,6 | AAB62297 | *P. putida* F1 |
| 08745 | MEG7359758 | - | cytochrome P450 | DitQ | 82,8 | AAR83738 | *P. abietaniphila* BKME-9 |
| 08750 | MEG7359759 | - | carboxymuconolactone decarboxylase family protein | DitP | 71,4 | AAR83739 | *P. abietaniphila* BKME-9 |
| 08755 | MEG7359760 | - | acetyl-CoA acetyltransferase | DitO | 82,6 | AAR83740 | *P. abietaniphila* BKME-9 |
| 08760 | MEG7359761 | - | 3-hydroxyacyl-CoA dehydrogenase | DitN | 81,7 | AAR83741 | *P. abietaniphila* BKME-9 |
| 08765 | MEG7359762 | - | fumarylacetoacetate hydrolase family protein | DitM | 81,2 | AAR83742 | *P. abietaniphila* BKME-9 |
| 08770 | MEG7359763 | - | amidohydrolase family protein | DitL | 77,5 | AAR83743 | *P. abietaniphila* BKME-9 |
| 08775 | MEG7359764 | - | ISL3 family transposase |  |  |  |  |
| 08780 | MEG7359765 | - | tetR family transcriptional regulator | DitK | 70 | AAR83744 | *P. abietaniphila* BKME-9 |
| 08785 | MEG7359766 | + | ATP-dependent acyl-CoA ligase | DitJ | 83,9 | AAD21073 | *P. abietaniphila* BKME-9 |
| 08790 | MEG7359767 | - | short-chain dehydrogenase/reductase family protein | DitI | 84,6 | AAD21071 | *P. abietaniphila* BKME-9 |
| 08795 | MEG7359768 | - | DUF2889 domain-containing protein |  |  |  |  |
| 08800 | MEG7359769 | - | diterpenoid dioxygenase beta-subunit | DitA2 | 85,6 | AAD21061 | *P. abietaniphila* BKME-9 |
| 08805 | MEG7359770 | - | diterpenoid dioxygenase alpha-subunit | DitA1 | 90,7 | AAD21063 | *P. abietaniphila* BKME-9 |
| 08810 | MEG7359771 | - | fumarylacetoacetate hydrolase family protein | DitH | 81,2 | AAD21070 | *P. abietaniphila* BKME-9 |
| 08815 | MEG7359772 | - | short-chain dehydrogenase/reductase family protein | DitG | 71 | AAD21069 | *P. abietaniphila* BKME-9 |
| 08820 | MEG7359773 | + | OB-fold domain-containing protein |  |  |  |  |
| 08825 | MEG7359774 | + | thiolase family protein | DitF | 89,4 | AAD21068 | *P. abietaniphila* BKME-9 |
| 08830 | MEG7359775 | - | IclR family transcriptional regulator | DitR | 71 | AAD21072 | *P. abietaniphila* BKME-9 |
| 08835 | MEG7359776 | - | MFS transporter | DitE | 63 | AAD21067 | *P. abietaniphila* BKME-9 |
| 08840 | MEG7359777 | - | fumarylacetoacetate hydrolase family protein | DitD | 70,8 | AAD21066 | *P. abietaniphila* BKME-9 |
| 08845 | MEG7359778 | - | aromatic diterpenoid extradiol ring-cleavage dioygenase | DitC | 84 | AAD21065 | *P. abietaniphila* BKME-9 |
| 08850 | MEG7359779 | - | short-chain dehydrogenase/reductase family protein | DitB | 80,2 | AAD21064 | *P. abietaniphila* BKME-9 |
| 08855 | MEG7359780 | - | dioxygenase DitA ferredoxin component | DitA3 | 77,5 | AAD21062 | *P. abietaniphila* BKME-9 |
| 08885 | MEG7359786 | + | diterpenoid dioxygenase alpha-subunit | DitA1 | 67,7 | AAD21063 | *P. abietaniphila* BKME-9 |
| 08890 | MEG7359787 | + | diterpenoid dioxygenase beta-subunit | DitA2 | 53,5 | AAD21061 | *P. abietaniphila* BKME-9 |
| 08895 | MEG7359788 | + | amidohydrolase family protein |  | 83,9 | AAR83737 | *P. abietaniphila* BKME-9 |
| 08900 | MEG7359789 | + | aromatic diterpenoid extradiol ring-cleavage dioygenase | DitC | 70,5 | AAD21065 | *P. abietaniphila* BKME-9 |
| 09170 | MEG7359842 | + | 3-hydroxyphenylacetate hydroxylase large subunit | MhaA | 72,4 | AAY16572 | *P. putida* U |
| 09175 | MEG7359843 | + | 3-hydroxyphenylacetate hydroxylase small subunit | MhaB | 58,3 | AAY16571 | *P. putida* U |
| 09310 | MEG7359870 | - | 4-hydroxyphenylacetate hydroxylase reductase | HpaC | 69,1 | ADA63517 | *P. putida* U |
| 09315 | MEG7359871 | - | 4-hydroxyphenylacetate hydroxylase oxygenase | HpaB | 83 | ADA63516 | *P. putida* U |
| 09320 | MEG7359872 | + | MFS transporter |  |  |  |  |
| 09325 | MEG7359873 | - | Bcr/CflA family multidrug efflux MFS transporter |  |  |  |  |
| 09330 | MEG7359874 | - | homoprotocatechuate degradation operon regulator | HpaA | 68,5 | ADA63526 | *P. putida* U |
| 09335 | MEG7359875 | - | hydroxyphenylacetate catabolism regulator | HpaY | 70 | ADA63527 | *P. putida* U |
| 09340 | MEG7359876 | + | 4-hydroxyphenylacetate degradation isomerase/decarboxylase | HpaG1 | 69,4 | ADA63525 | *P. putida* U |
| 09345 | MEG7359877 | + | 4-hydroxyphenylacetate degradation isomerase/decarboxylase | HpaG2 | 86,5 | ADA63524 | *P. putida* U |
| 09350 | MEG7359878 | + | 5-carboxymethyl-2-hydroxymuconic semialdehyde dehydrogenase | HpaE | 91,6 | ADA63523 | *P. putida* U |
| 09355 | MEG7359879 | + | homorotocatechuate 2,3-dioxygenase | HpaD | 86,1 | ADA63522 | *P. putida* U |
| 09360 | MEG7359880 | + | 5-carboxymethyl-2-hydroxymuconate isomerase | HpaF | 28,6 | ADA63521 | *P. putida* U |
| 09365 | MEG7359881 | + | MFS transporter | HpaX | 73,9 | ADA63520 | *P. putida* U |
| 09370 | MEG7359882 | + | 2-oxo-hepta-3-ene-1,7-dioate hydratase | HpaH | 89,1 | ADA63519 | *P. putida* U |
| 09375 | MEG7359883 | + | 2,4-dihydroxyhept-2-ene-1,7-dioate aldolase | HpaI | 82,8 | ADA63518 | *P. putida* U |
| 14785 | MEG7360948 | - | AraC family transcriptional regulator |  | 69,4 | AAG05899 | *P. aeruginosa* PAO1 |
| 14790 | MEG7360949 | + | anthranilate_1_2-dioxygenase alpha-subunit | AntA | 84,2 | AAG05900 | *P. aeruginosa* PAO1 |
| 14795 | MEG7360950 | + | anthranilate_1_2-dioxygenase beta-subunit | AntB | 77,9 | AAG05901 | *P. aeruginosa* PAO1 |
| 14800 | MEG7360951 | + | anthranilate_1_2-dioxygenase reductase | AntC | 73 | AAG05902 | *P. aeruginosa* PAO1 |
| 14835 | MEG7360958 | - | aminocarboxymuconate-semialdehyde decarboxylase | NbaD | 68,7 | BAC65312 | *P. fluorescens* KU-7 |
| 14840 | MEG7360959 | - | 3-hydroxyanthranilate 3,4-dioxygenase | NbaC | 63,5 | BAC65311 | *P. fluorescens* KU-7 |
| 14845 | MEG7360960 | - | 2-aminomuconate deaminase | NbaF | 77,5 | BAC65310 | *P. fluorescens* KU-7 |
| 14850 | MEG7360961 | - | 4-oxalocrotonate decarboxylase | NbaG | 65,5 | BAC65309 | *P. fluorescens* KU-7 |
| 14855 | MEG7360962 | - | 4-hydroxy-2-oxovalerate aldolase | NbaI | 82,2 | BAC65308 | *P. fluorescens* KU-7 |
| 14860 | MEG7360963 | - | acetaldehyde dehydrogenase (acetylating) | NbaJ | 69,3 | BAC65307 | *P. fluorescens* KU-7 |
| 14865 | MEG7360964 | - | 2-keto-4-pentenoate hydratase | NbaH | 64,5 | BAC65306 | *P. fluorescens* KU-7 |
| 14870 | MEG7360965 | - | 2-hydroxymuconic semialdehyde dehydrogenase | NbaE | 69,2 | BAC65304 | *P. fluorescens* KU-7 |
| 14875 | MEG7360966 | - | outer membrane porin | NicP-I | 74,1 | AAN68776 | *P. putida* KT2440 |
| 14880 | MEG7360967 | - | benzoate transporter | BenE | 65,5 | AAN68775 | *P. putida* KT2440 |
| 14885 | MEG7360968 | - | catechol 1,2-dioxygenase | CatA | 80,3 | AAN68774 | *P. putida* KT2440 |
| 14890 | MEG7360969 | - | muconolactone isomerase | CatC | 92,7 | AAN69311 | *P. putida* KT2440 |
| 14895 | MEG7360970 | - | muconate cycloisomerase | CatB | 85 | AAN69312 | *P. putida* KT2440 |
| 14900 | MEG7360971 | + | LysR family transcriptional regulator | CatR | 81,5 | AAN69313 | *P. putida* KT2440 |
| 14905 | MEG7360972 | - | outer membrane porin |  |  |  |  |
| 14910 | MEG7360973 | - | amidase |  |  |  |  |
| 14915 | MEG7360974 | - | benzoate MFS transporter | BenK | 77,4 | AAN68773 | *P. putida* KT2440 |
| 14920 | MEG7360975 | - | 1,6-dihydroxycyclohexa-2,4-diene-1-carboxylate dehydrogenase | BenD | 81,6 | AAN68772 | *P. putida* KT2440 |
| 14925 | MEG7360976 | - | benzoate 1,2-dioxygenase reductase | BenC | 84,2 | AAN68771 | *P. putida* KT2440 |
| 14930 | MEG7360977 | - | benzoate 1,2-dioxygenase betal subunit | BenB | 87,6 | AAN68770 | *P. putida* KT2440 |
| 14935 | MEG7360978 | - | benzoate 1,2-dioxygenase alpha subunit | BenA | 85,6 | AAN68769 | *P. putida* KT2440 |
| 14940 | MEG7360979 | - | benABC operon transcriptional activator BenR | BenR | 70,4 | AAN68767 | *P. putida* KT2440 |
| 15845 | MEG7361157 | - | phenol/hydroxyquinone monooxygenase | MhqA | 59,7 | BAE46529 | *Burkholderia* sp. NF100 |
| 16355 | MEG7361255 | - | FAD-dependent monooxygenase | MhqA | 40,9 | BAE46529 | *Burkholderia* sp. NF100 |
| 16365 | MEG7361256 | - | 2,3-Dihydroxybiphenyl dioxygenases and enzymes of methylhydroquinone metabolism | MhqB | 60,2 | BAE46530 | *Burkholderia* sp. NF100 |
| 16370 | MEG7361257 | - | fumarylacetoacetate hydrolase family protein |  |  |  |  |
| 16545 | MEG7361292 | + | resorcinol 4-monooxygenase |  | 57.3/26.4 | CAF19862/ ACN43577 | *C. glutamicum* ATCC13032/*P. putida* DLL-E4 |
| 16550 | MEG7361293 | + | maleylacetate reductase |  | 41.4/55.4 | CAF19863/ ACN43572 | *C. glutamicum* ATCC13032/*P. putida* DLL-E4 |
| 16555 | MEG7361294 | + | hydroxyquinol 1,2-dioxygenase |  | 48/57 | CAF19864/ ACN43573 | *C. glutamicum* ATCC13032/*P. putida* DLL-E4 |
| 17940 | MEG7361568 | - | catechol 1,2-dioxygenase | CatA | 75,7 | AAN68774 | *P. putida* KT2440 |
| 17945 | MEG7361569 | - | NtrC family transcriptional regulator | DmpR | 65,4 | BAP28465 | *P. putida* CF600 |
| 17950 | MEG7361570 | + | phenol hydroxylase subunit DmpK | DmpK | 68,1 | BAP28466 | *P. putida* CF600 |
| 17955 | MEG7361571 | + | phenol hydroxylase subunit DmpL | DmpL | 64,9 | BAP28467 | *P. putida* CF600 |
| 17960 | MEG7361572 | + | phenol hydroxylase subunit DmpM | DmpM | 77,3 | BAP28468 | *P. putida* CF600 |
| 17965 | MEG7361573 | + | phenol hydroxylase subunit DmpN | DmpN | 84,7 | BAP28469 | *P. putida* CF600 |
| 17970 | MEG7361574 | + | phenol hydroxylase subunit DmpO | DmpO | 56,4 | BAP28470 | *P. putida* CF600 |
| 17975 | MEG7361575 | + | phenol hydroxylase subunit DmpP | DmpP | 85 | BAP28471 | *P. putida* CF600 |
| 18285 | MEG7361629 | - | 4-hydroxy-2-oxovalerate aldolase |  | 100 | ANI15724 | *P. citronellolis* SJTE-3 |
| 18290 | MEG7361630 | - | acetaldehyde dehydrogenase (acetylating) |  | 99,7 | ANI15724 | *P. citronellolis* SJTE-3 |
| 18295 | MEG7361631 | - | 2-keto-4-pentenoate hydratase |  | 99,1 | ANI18514 | *P. citronellolis* SJTE-3 |
| 18300 | MEG7361632 | - | orcinol hydroxylase |  | 100 | AAQ10529 | *P. putida* ORC |
| 18305 | MEG7361633 | - | extradiol dioxygenase related to those involved in orcinol degradation |  | 100 | AAQ10528 | *P. putida* ORC |
| 18310 | MEG7361634 | - | 2-hydroxy-6-ketonona-2,4-dienedioic acid hydrolase |  | 100 | AAQ10527 | *P. putida* ORC |
| 18450 | MEG7361662 | + | 2-oxopent-4-enoate hydratase | DmpE | 63 | BAP28476 | *P. putida* CF600 |
| 18455 | MEG7361663 | + | 4-oxalocrotonate decarboxylase | DmpH | 62,3 | BAP28479 | *P. putida* CF600 |
| 18460 | MEG7361664 | + | 4-oxalocrotonate tautomerase | DmpI | 48,3 | BAP28480 | *P. putida* CF600 |
| 18465 | MEG7361665 | + | acetaldehyde dehydrogenase (acetylating) | DmpF | 70,4 | BAP28477 | *P. putida* CF600 |
| 18470 | MEG7361666 | + | XylT type ferredoxin | DmpQ | 49,4 | BAP28472 | *P. putida* CF600 |
| 18475 | MEG7361667 | + | catechol 2,3-dioxygenase | DmpB | 66,1 | BAP28473 | *P. putida* CF600 |
| 18480 | MEG7361668 | + | 4-hydroxy-2-oxovalerate aldolase | DmpG | 89,2 | BAP28478 | *P. putida* CF600 |
| 19150 | MEG7361796 | - | PaaN ring-opening enzyme | PaaN | 84,6 | AAC24340 | *P. putida* U |
| 19155 | MEG7361797 | - | PaaK ring-oxidation complex; protein 5 | PaaK | 80,7 | AAC24337 | *P. putida* U |
| 19160 | MEG7361798 | - | PaaJ ring-oxidation complex; protein 4 | PaaJ | 82,9 | AAC24336 | *P. putida* U |
| 19165 | MEG7361799 | - | PaaI ring-oxidation complex; protein 3 | PaaI | 74,9 | AAC24335 | *P. putida* U |
| 19170 | MEG7361800 | - | PaaH ring-oxidation complex; protein 2 | PaaH | 91,4 | AAN04575 | *P. putida* U |
| 19175 | MEG7361801 | - | PaaG ring-oxidation complex; protein 1 | PaaG | 92,6 | AAC24334 | *P. putida* U |
| 24185 | MEG7362785 | + | Rieske alpha subunit similar to those involved in indole acetic acid degradation and related enzymes | IacC | 64,2 | ABY62759 | *P. putida* 1290 |
| 24190 | MEG7362786 | + | Rieske beta subunit | IacD | 39,2 | ABY62760 | *P. putida* 1290 |
| 24195 | MEG7362787 | + | short chain dehydrogenase | IacE | 54,5 | ABY62761 | *P. putida* 1290 |
| 24200 | MEG7362788 | + | Rieske enzyme reductase subunit | IacF | 41,8 | ABY62762 | *P. putida* 1290 |
| 24205 | MEG7362789 | - | DNA-binding response regulator |  | 46,1 | QCI12930 | *P. putida* 1290 |
| 24210 | MEG7362790 | + | flavin reductase | IacG | 54,2 | ABY62763 | *P. putida* 1290 |
| 24545 | MEG7362857 | - | IclR family regulator | MhpR | 69,4 | WP_001550698 | *E. coli* K-12 |
| 24550 | MEG7362858 | + | 3-(3-hydroxy-phenyl)propionate hydroxylase | MhpA | 70,5 | CAA70747 | *E. coli* K-12 |
| 24555 | MEG7362859 | + | 2,3-Dihydroxyphenylpropionate dioxygenase | MhpB | 75 | CAA70748 | *E. coli* K-12 |
| 24560 | MEG7362860 | + | 2-hydroxy-6-oxo-6-phenylhexa-2,4-dienoate hydrolase | MhpC | 69,9 | CAA70749 | *E. coli* K-12 |
| 24565 | MEG7362861 | + | 2-keto-4-pentenoate hydratase | MhpD | 71,4 | CAA70750 | *E. coli* K-12 |
| 24570 | MEG7362862 | + | acetaldehyde dehydrogenase (acetylating) | MhpF | 80,4 | CAA70751 | *E. coli* K-12 |
| 24575 | MEG7362863 | + | 4-hydroxy-2-oxovalerate aldolase | MhpE | 92,6 | CAA70752 | *E. coli* K-12 |
| 24580 | MEG7362864 | + | 3-hydroxyphenylpropionate transporter | MhpT | 69,1 | WP_000107624 | *E. coli* K-12 |
| 28515 | MEG7363636 | - | fumarylacetoacetase | HmgB | 69,8 | AAO12528 | *P. putida* U |
| 28520 | MEG7363637 | - | homogentisate 1,2-dioxygenase | HmgA | 75,2 | AAO12527 | *P. putida* U |
| 28525 | MEG7363638 | + | IclR family transcriptional regulator | HmgR | 66,8 | AAO12526 | *P. putida* U |
| 28530 | MEG7363639 | - | 4-hydroxyphenylpyruvate dioxygenase | Hpd | 90,2 | AAO12525 | *P. putida* U |
| 28535 | MEG7363640 | - | maleylacetoacetate isomerase | HmgC | 74,6 | AAO12529 | *P. putida* U |
| 28540 | MEG7363641 | - | 4-hydroxybenzoate 3-monooxygenase | PobA | 75,4 | AAN69138 | *P. putida* KT2440 |
| 28545 | MEG7363642 | + | AraC family transcriptional regulator | PobR | 50,4 | AAN69139 | *P. putida* KT2440 |
| 28550 | MEG7363643 | + | LysR family transcriptional regulator PcaQ | PcaQ | 66,1 | AAN67334 | *P. putida* KT2440 |
| 28555 | MEG7363644 | + | protocatechuate 3,4-dioxygenase beta subunit | PcaH | 90,3 | AAN70229 | *P. putida* KT2440 |
| 28560 | MEG7363645 | + | protocatechuate 3,4-dioxygenase alpha subunit | PcaG | 80,6 | AAN70228 | *P. putida* KT2440 |
| 28565 | MEG7363646 | + | 3-carboxy-*cis,cis*-muconate cycloisomerase | PcaB | 75 | AAN67002 | *P. putida* KT2440 |
| 28570 | MEG7363647 | + | 4-carboxymuconolactone decarboxylase | PcaC | 86,7 | AAN67004 | *P. putida* KT2440 |
| 28695 | MEG7363670 | + | benzaldehyde dehydrogenase | PchA | 78,4 | AGO01127 | *P. putida* NCIMB 9866 |
| 28700 | MEG7363671 | + | *p*-cresol methylhydroxylase cytochrome subunit | PchC | 50,4 | AGO01128 | *P. putida* NCIMB 9866 |
| 28705 | MEG7363672 | + | hypothetical protein | PchX | 47,8 | AGO01129 | *P. putida* NCIMB 9866 |
| 28710 | MEG7363673 | + | *p*-cresol methylhydroxylase flavoprotein subunit | PchF | 79,3 | AGO01130 | *P. putida* NCIMB 9866 |

**Table S5**: Benzoate transformation rates of glucose-grown resting cells of EB200 wild type and mutant strains during the first 60 minutes of the conversion experiments depicted in Fig. S4 and Fig. 3.

| **Cells** | **OD_546_** | **transformation rate**  **(µmol L^-1^ h^-1^ OD_546_^-1^)** |
| --- | --- | --- |
| EB200 wild type | 8.7 | 216 |
| EB200 ∆*cmtB* | 8.8 | 239 |
| EB200 ∆*benA* ∆*cmtB* | 10.2 | 147 |

**Table S6:** Yields of individual steps and total yield of the optimized production protocol for the 2,3-DD sodium salt monohydrate

| **Protocol steps** | **Yield [%]** |
| --- | --- |
| Biotransformation | 98 |
| 4-fold extraction with BuOH | 77 |
| Re-extraction of BuOH phase with dilute NaOH | 98 |
| Precipitation with EtOH:concentrate = 9:1 | 81 – 92 |
| Total yield | 60 – 68 |

**Table S7**: Transformation of benzoates by 4-ethylbenzoate-grown resting cells of strain EB200 at an OD_546_ of 3.0. Transformation was monitored for 60 minutes after addition of the substrate

| **Substrates (5 mM)** | **transformation rate**  **(µmol L^-1^ h^-1^ OD_546_^-1^)** |
| --- | --- |
| benzoate | 242 |
| 4-methylbenzoate | 839 |
| 4-ethylbenzoate | 999 |
| *p*-cumate | 445 |

**Table S8**: NCBI Genbank locus tags for genes of the upper *p*-cumate and benzoate degradative pathways in EB200, accession JBAISM01

| **Locus tag** | **Gene** | **Description** |
| --- | --- | --- |
| V5264_07910 | *cmtAa* | PCDO ferredoxin reductase |
| V5264_07905 | *cmtAb* | PCDO large subunit |
| V5264_07900 | *cmtAc* | PCDO small subunit |
| V5264_07895 | *cmtC* | 2,3-dihydroxy-*p*-cumate 3,4-dioxygenase |
| V5264_07890 | *cmtB* | 2,3-dihydroxy-2,3-dihydro-*p*-cumate dehydrogenase |
| V5264_07885 | *cmtAd* | PCDO ferredoxin |
| V5264_07880 | *cmtD* | 2-hydroxy-3-carboxy-6-oxo-7-methylocta-2,4-dienoate decarboxylase |
| V5264_14935 | *benA* | benzoate 1,2-dioxygenase large subunit |
| V5264_14930 | *benB* | benzoate 1,2-dioxygenase small subunit |
| V5264_14925 | *benC* | benzoate 1,2-dioxygenase reductase |
| V5264_14920 | *benD* | 1,2-dihydroxy-2-hydrobenzoate dehydrogenase |

**Appendix I - Extraction procedures and efficiencies**

Crude fermentation supernatants containing 5 mM of 2,3-DD were saturated with NaCl, acidified with HCl to a pH of 2 – 3 in an ice bath, and then quickly extracted with equal volumes of ethyl acetate (EtOAc) or 1-butanol (BuOH). After separation of the aqueous and organic phases by centrifugation, the concentration of 2,3-DD was determined. The resulting BuOH phase containing ca. 2 mM 2,3-DD was re-extracted with diluted NaOH to determine the extraction coefficient at pH 7. Phosphate was determined according to the stannous chloride method (Greenberg *et al.*, 1992) after extraction of 20x concentrated phosphate buffer (20 g/L KH_2_PO_4_ and 70 g/L Na_2_HPO_4_ · 2 H_2_O) with BuOH. As shown in Table S9, pH in the given range had a minor impact on extraction efficiency of 2,3-DD, but a larger effect on the unwelcome extraction of phosphate species from the fermentation buffer. Considerably higher extraction efficiencies for 2,3-DD were obtained with BuOH compared to EtOAc as the organic phase. Omission of NaCl resulted in a high carryover of water and phosphates into the organic phase.

**Table S9**: Extraction coefficients for 2,3-DD and phosphate. Aqueous phases (aq) were saturated with NaCl prior to extraction if not specified otherwise. n.d., not determined.

| **Solvent system** | **pH** | **Extraction coefficients c_org_/c_aq_** | |
| --- | --- | --- | --- |
|  |  | **2,3-DD** | **Phosphate** |
| EtOAc/aq | 2 | 0.10 | n.d. |
| BuOH/aq, no NaCl | 2 | 0.60 | 0.026 |
| BuOH/aq | 2 | 0.70 | 0.007 |
| BuOH/aq | 2.5 | 0.68 | 0.003 |
| BuOH/aq | 3 | 0.56 | 0.001 |
| BuOH/aq, no NaCl | 7 | <0.01 | n.d. |

**Appendix II - Optimized protocol for production of the *cis*-2,3-DD sodium salt monohydrate**

500 mL autoinduction cultures of freshly transformed BL21(DE3) pT7-5(EB200_cmtA) were grown at 25 °C in continuously stirred 5 L bottles until stagnation of growth was observed (1-2 days). Substrate transformation was carried out in 500 mL shaking flasks containing 100 mL cell suspension with an OD_546_ of ca. 20 in PB in a fed-batch process at 30 °C with initial concentrations of 5 mM benzoate and 5 mM glucose. Benzoate and glucose were further added at 5 mM steps as indicated by HPLC monitoring of benzoate conversion. After conversion of each 10 mM of benzoate, the culture was transferred to a fresh shaking flask to ensure oxygen availability. After transformation for 5-6 hours, cells were separated from the culture supernatant, stored overnight on a rotary shaker in 50 mL PB supplemented with 10 mM glucose, and reused on the following day either with fresh PB or with the fermentation supernatant from the previous day to transform any residual benzoate.

Crude fermentation supernatants containing 20-30 mM of 2,3-DD were reduced to 1/10-1/20 of their volume in a rotational evaporator, saturated with NaCl, acidified with conc. HCl to pH 2-2.5 at 0 °C, and extracted four times with equal volumes of BuOH. The undried organic phases were combined, and re-extracted and neutralized with half their volume of diluted NaOH. The aqueous phase was concentrated to 500-700 mM in a rotary evaporator with the bath temperature set to 55 °C, mixed with ethanol at a ratio of EtOH:concentrate = 9:1, and left overnight at room temperature to precipitate the sodium salt monohydrate of 2,3-DD. The supernatant was carefully removed with a pipette and the crystals were dried over silica gel.

The total yield of this optimized protocol was 60-68 %. A graphical chart of the process is presented in Fig. S15.


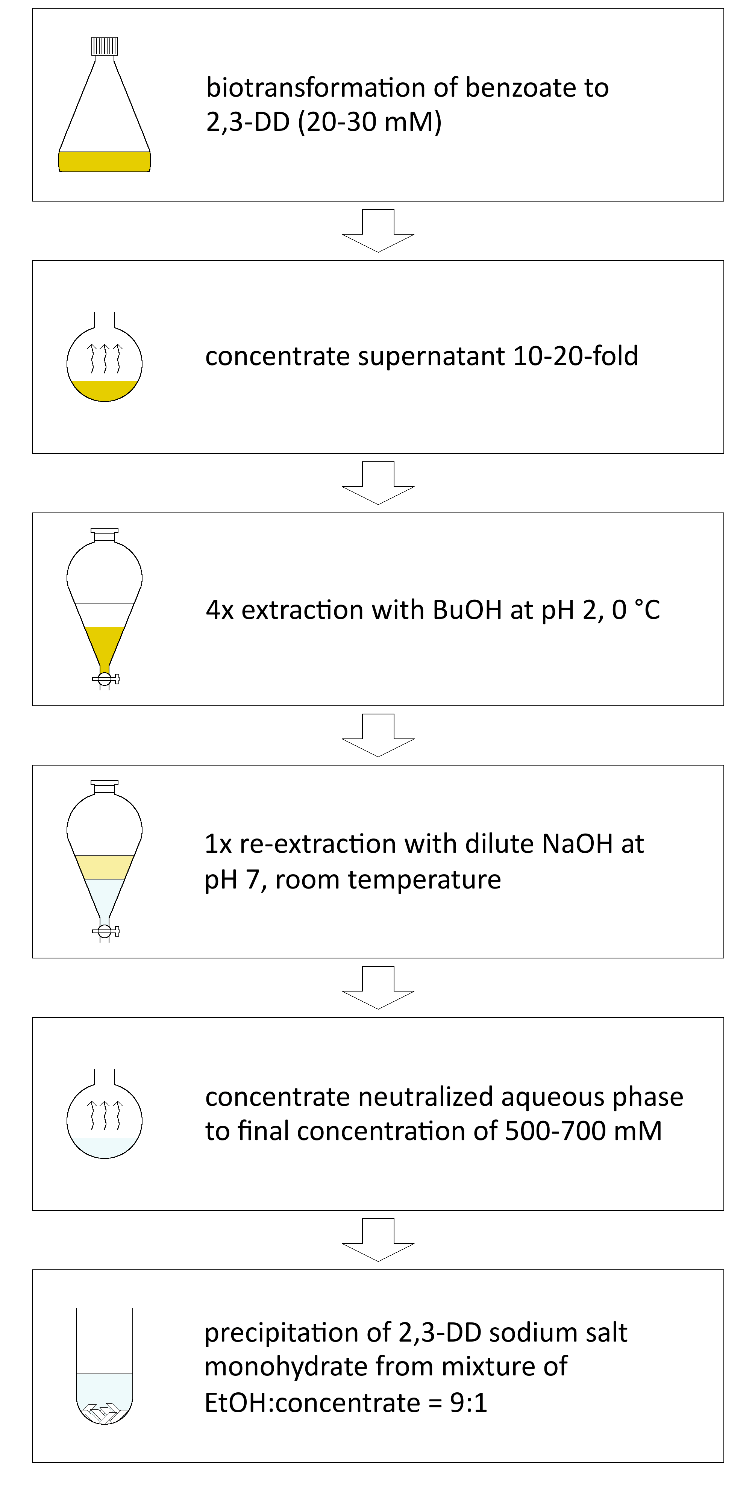


**Fig. S15**: Flowchart of the production process for the 2,3-DD sodium salt monohydrate.

**List of abbreviations**

1,2-DD (1*S*,2*R*)-1,2-dihydroxy-2-hydrobenzoate

2,3-DD (2*R*,3*S*)-2,3-dihydroxy-2,3-dihydrobenzoate

2,3-DHB 2,3-dihydroxybenzoate

4-EB 4-ethylbenzoate

4-IB 4-isopropylbenzoate, *p*-cumate

4-MB 4-methylbenzoate

BPDO biphenyl 2,3-dioxygenase

BuOH 1-butanol

BZDO benzoate 1,2-dioxygenase (BenABC)

CDO chlorobenzene dioxygenase

EtOAc ethyl acetate

EtOH ethanol

iPrOH isopropyl alcohol

NDO naphthalene 1,2-dioxygenase

PB phosphate buffer (1 g L^-1^ KH_2_PO_4_, 3.5 g L^-1^ Na_2_HPO_4_ · 2 H_2_O; pH 7.1)

PCDO *p*-cumate 2,3-dioxygenase (CmtAaAbAcAd)

RO Rieske non-heme iron oxygenase

TDO toluene 2,3-dioxygenase

**References**

Aoki, H., Kimura, T., Habe, H., Yamane, H., Kodama, T., and Omori, T. (1996) Cloning, Nucleotide Sequence, and Characterization of the Genes Encoding Enzymes Involved in the Degradation of Cumene to 2-Hydroxy-6-Oxo-7-Methylocta-2,4-Dienoic Acid in *Pseudomonas fluorescens* IP01. *J Ferment Bioeng* **81** (3): 187–196.

Boyd, D.R., Blacker, J., Byrne, B., Dalton, H., Hand, M.V., Kelly, S.C., *et al.* (1994) Acid-catalysed Aromatisation of Benzene *cis*-1,2-dihydrodiols: a Carbocation Transition State poorly stabilised by Resonance. *J Chem Soc, Chem Commun* (3): 313.

Castillo, A.M., Patiny, L., and Wist, J. (2011) Fast and accurate algorithm for the simulation of NMR spectra of large spin systems. *J Magn Reson* **209** (2): 123–130.

Eaton, R.W. (1996) *p*-Cumate Catabolic Pathway in *Pseudomonas putida* F1: Cloning and Characterization of DNA Carrying the *cmt* Operon. *J Bacteriol* **178** (5): 1351–1362.

Greenberg, A.E., Clesceri, L.S., and Eaton, A.D. (1992) *Standard methods for the examination of water and wastewater.* Washington, D.C.: American Public Health Association.

Platt, R., Drescher, C., Park, S.K., and Phillips, G.J. (2000) Genetic System for Reversible Integration of DNA Constructs and *lacZ* Gene Fusions into the *Escherichia coli* Chromosome. *Plasmid* **43** (1): 12–23.

Schäfer, A., Tauch, A., Jäger, W., Kalinowski, J., Thierbach, G., and Pühler, A. (1994) Small mobilizable multi-purpose cloning vectors derived from the *Escherichia coli* plasmids pK18 and pK19: Selection of defined deletions in the chromosome of *Corynebacterium glutamicum*. *Gene* **145** (1): 69–73.

Simon, R., Priefer, U., and Pühler, A. (1983) A Broad Host Range Mobilization System for In Vivo Genetic Engineering: Transposon Mutagenesis in Gram Negative Bacteria. *Nat Biotechnol* **1** (9): 784–791.

Studier, F.W., and Moffatt, B.A. (1986) Use of Bacteriophage T7 RNA Polymerase to Direct Selective High-level Expression of Cloned Genes. *J Mol Biol* **189** (1): 113–130.

Suen, W.-C. (1991) *Gene expression of naphthalene dioxygenase from Pseudomonas sp. NCIB 9816-4 in Escherichia coli.* Iowa City, Iowa, USA.

Yanisch-Perron, C., Vieira, J., and Messing, J. (1985) Improved M13 phage cloning vectors and host strains: nucleotide sequences of the M13mp18 and pUC19 vectors. *Gene* **33** (1): 103–119.
